# Using participatory action research to reimagine community mental health services in Colombia: a mixed-method study protocol

**DOI:** 10.1136/bmjopen-2022-069329

**Published:** 2022-12-21

**Authors:** Rochelle Burgess, María Cecilia Dedios Sanguineti, Darío Maldonado-Carrizosa, Laura Fonseca, Norha Vera San Juan, Diego Lucumí, Mónica González-Gort, Mónica Carreño Melgar, María Fanny Gaviria, Diego Ferney Tovar, Sandra Jovchelovitch

**Affiliations:** 1 UCL Institute for Global Health, London, UK; 2 School of Government, Universidad de los Andes, Bogota, Colombia; 3 Department of Psychological and Behavioural Science, London School of Economics and Political Science, London, UK; 4 Rapid Research Appraisal and Evaluation Lab, University College London, London, UK; 5 Corporación Manigua, Florencia, Colombia; 6 Cooperativa Multiactiva Para El Buen Vivir Y La Paz Del Caquetá, Florencia, Colombia

**Keywords:** MENTAL HEALTH, Depression & mood disorders, PUBLIC HEALTH

## Abstract

**Introduction:**

Mental healthcare systems are challenged by how they hear and respond to what marginalised communities experience as drivers of mental distress. In Colombia, this challenge intersects with wider challenges facing post-conflict reconstruction. Our pilot study will explore the feasibility and acceptability of a participatory approach to developing community-led participatory interventions for community mental health systems strengthening and mental health improvement, in two sites in Caquetá, Colombia.

**Methods and analysis:**

The project is divided into three distinct phases aligned with community participatory action research cycles: diagnostic, intervention and evaluation. This allows us to use a participatory approach to design a community-led, bottom-up intervention for mental health systems strengthening and the promotion of mental health and well-being.

The diagnostic phase explores local understandings of mental health, mental distress and access to mental health services from community members and health providers. The intervention stage will be guided by a participatory Theory of Change process. Community priorities will inform the development of a participatory, learning and action (PLA) informed group intervention, with a community linkage forum. The pilot of the PLA intervention will be evaluated using MRC process evaluation guidelines.

**Ethics and dissemination:**

This project has received ethical approval from two sources. Universidad de Los Andes (2021–1393) and the University College London (16127/005). Dissemination of findings will include academic publications, community forums, policy briefs and visual media (cartoons, pod casts and short films).

**Strengths and limitations of this study**

- This pilot study aims to provide evidence for a new methodology that meaningfully involves citizens developing and strengthening mental health systems in complex settings.
- The study pilots for the first time in Colombia participatory action research to design participatory learning and action groups (PLA) for improving mental health and strengthening community mental health systems.
- PLA groups will enable better collaboration between community knowledge systems, community members and the services that are designed to support them, through ‘community link’ activities.
- The main challenge facing this pilot is the integration of participation across multiple sectors.
- Participatory action research processes can be directly impacted by wider geopolitical realities—such as the UK government funding cuts, which disrupted community processes and relationship building.

## Introduction

Globally, the burden of mental health conditions is shaped by gaps in services. In low-middle income countries, 75% of the population lacks access to any form of care.^1^ The COVID-19 pandemic exacerbated these challenges as intersecting social realities deepen distress, increase the incidence of mental health disorders and overburden health systems.^2^ In the case of Colombia, political violence, poverty and displacement further aggravate this burden. Previous research shows that victims of armed conflict are more likely to suffer from mental health disorders,^3^ with poverty explaining 86% of mental health inequalities in the country.^4^

Six years after the Peace Accords between the Colombian Government and the FARC-EP (Fuerzas Armadas Revolucionarias de Colombia - Ejército del Pueblo, in Spanish) guerrilla, there are still barriers in the implementation of the Psychosocial Care and Comprehensive Health Services for Victims programme and the Psychosocial Wellbeing Component in the reintegration route for ex-combatants (Resolution n. 4309). In the case of ex-combatants, a dual status of victims and perpetrators requires balancing psychosocial well-being, personal protection and political acceptability of mental health services. This population, like the victims of the conflict, reside in rural areas where services are scarce or non-existent.^3 5^

Mental-health care systems are challenged by how they hear and respond to what marginalised communities experience as drivers of mental distress.^6–8^ This is acknowledged by global^9^ and national priorities, which call for providing accessible and quality services to overlooked communities. In Colombia, this includes territories and rural populations (campesinos) that are the focus of Territorially Focused Development Programmes (PDETs in Spanish), a national programme of development prioritising those who have been heavily affected by disproportionate armed conflict, poverty, illicit economies and institutional fragility.^10 11^

Scaling-up services is important but only a partial response; sustainable solutions to improve mental health require dialogue between health systems and communities.^12^ Community-owned and anchored interventions are critical to re-establishing trust between local populations and systems, particularly after periods of extended upheaval. In this context, integrating community-level experiences of mental health and mental distress with institutional responses by state-level actors is a necessary step towards effective community mental health services. This requires a multilevel interdisciplinary perspective that links individual and community well-being to wider institutional, socioeconomic and political contexts. Community participatory action research (CPAR) approaches allow us to explore the ability to identify strengths and solutions produced by communities for communities, connecting them to wider systems, while acknowledging them as agents with the capacity to create effective, context-sensitive solutions.^13^

As Colombia begins to refocus its efforts towards achieving these global and national policy aims, three critical areas require attention: (1) wider social and political contextual factors that drive experiences of poor mental health,^14^ (2) increasing understanding of local embodied knowledge and lived experiences of communities and their relevance for building knowledge about mental health,^15^ and (3) the role and resources offered by community participation in the codesign of interventions and services that are effective.^8^

In response to these demands, we will implement a participatory process to design, implement and evaluate a participatory intervention to strengthen community mental healthcare systems in two PDET communities in Caquetá-Colombia. We are guided by the following research question: *what are the pathways, mechanisms, and resources needed to catalyse collaborative action between communities and institutions for promoting and improving mental health services for PDET communities?* To this end, we aim:

1. To co-design and co-implement a participatory group intervention to create trust and opportunities for collaborative action between community and health system actors to improve the performance of community mental health services.
2. To co-evaluate the group intervention in terms of process, outcomes (including individual and community mental health) and simulations of the cost-benefit and cost-effectiveness of the intervention at individual, community and health services levels.
3. To produce a manual based on the development, implementation and evaluation of the intervention to guide communities and institutions in the application of these methods for developing and scaling up community mental health services in Colombia. We expect these tools to be made widely applicable in other low-resource or conflict-affected settings.

The project is divided into distinct phases aligned with CPAR cycles reflecting diagnostic, intervention and evaluation. This protocol presents the STARS-C (Starting from the bottom: Using Participatory Action Research to re-imagine local mental health services in Colombia) objectives, procedures and methodological considerations for implementing a participatory mental health research project in conflict areas amidst the COVID-19 pandemic.

## Methods and analysis

The project will be implemented in inter-related phases aligned with participatory action research (PAR). It will run from February 2021 to May 2023 in Caquetá, Colombia. Implementation of the group intervention will run from July 2022 to March 2023. The project has been co-designed through existing partnerships involving academics and two community-based organisations: (1) the Manigua Corporation (*Corpomanigua*), an organisation of women with experience in the design and implementation of projects with marginalised communities, located in Florencia, representing an urban community and (2) the Multi-active Cooperative for Wellbeing and Peace of Caquetá (*Cooperativa Multiactiva para el Buen Vivir y la Paz del Caquetá*), which represents a rural community of ex-combatants from the former guerrilla FARC-EP, located in the small village Héctor Ramírez Poblado Center (CP-HR: former Territorial Space for Training and Reincorporation Héctor Ramírez) in the municipality of La Montañita.

Co-design and coimplementation will be further achieved through the appointment of community researchers (two from each site), who live and work in the communities being studied. They will be involved in all stages of the implementation of the project as detailed below and were appointed prior to the drafting of this protocol. To ensure more equal partnerships in this work, community researchers were trained in collecting qualitative information, quantitative questionnaires and in psychological first aid to support potential psychological and emotional distress among participants. Regular supervision is provided in real-time planned meetings. WhatsApp groups are used for constant communication.

### Setting

Caquetá is one of Colombia’s 32 departments, and the only region of the country in which all municipalities are included in the Territorially Focused Development Plans (PDET in Spanish). The project will be conducted in two of these PDET municipalities: Florencia and La Montañita. Each of the municipalities also represents diversity within a more general context of deprivation and adversity.

Florencia is Caquetá’s capital city and constitutes its largest population with 173 011 inhabitants.^16^ Updated mental health statistics are not available at the municipality level; however, a report by MSF (2010) in Caquetá suggests that of the 60% of the nearly 5000 patients affected by armed conflict and internal displacement, 18% were diagnosed with adaptative disorders, 18% with relationship problems and problems associated with abuse or neglect, 11% by major depression with one episode, 9% with grief and 8% with mood disorders.^17^ Arguably, the prevalence of these mental health disorders relates to structural drivers such as high unemployment levels. According to the latest report done by the National Administrative Department of Statistics in 2020, the unemployment rate in Florencia was 25%, with women having a higher unemployment rate (29.2%) than men (21.5%),^16^ both much more, than the current unemployment national rate of 11%.^18^ As an urban area, Florencia has access to some specialised mental health facilities and staff, including psychologists, psychiatrists and nurses.

La Montañita is a rural area located to the south-west of Florencia and one of the areas most affected by the armed conflict, with 8756 victims out of a total of 14 692 inhabitants.^16 19^ No mental health statistics are available for the municipality but reports from local organisations point to mental distress associated with poverty and conflict as well unmet care needs. The project will be carried out in a small village self-named *Centro Poblado Héctor Ramirez,* which is one of theFormerTerritorial Spaces for Training and Reincorporation for former FARC-EP combatants (AETCR in Spanish) in La Montanita.

### Design

The STARS-C project outlines a three-phase process to guide stakeholders in the development and strengthening of community led mental health systems. It is informed by co-production principles, to enable a platform for involving community members in a process of thinking through what changes are needed to improve access to, and the quality of mental health services.^20^ Co-production principles demand the inclusion of everyday actors, or potential service users, within processes of design and development. We will achieve this through involving everyday community members using a CPAR^21^ model, to think through what changes are needed to improve access to and quality of mental health services.^20^ As such the project combines participatory qualitative inquiry across its three phases of diagnosis, intervention and evaluation (see table 1) with quantitative assessments of mental health outcomes in a process described below.

**Table 1:** Phases and data collection strategies

Phase	Data collection	Participants	
		La Montañita	Florencia
Diagnostic	Focus group 1: local understandings of mental health and mental distress—Tree of Life	n=42	n=57
	Focus group 2: evaluation of standardised measures of mental health	n=34	n=49
	Interviews health providers	n=13	n=17
	Whatsapp focus groups health providers	n=11	n=10
	Motivated ethnography (1 month)	Local Hospital-Community health post	City Hospital
Intervention Design	Theory of change workshop	N=25	n=25
Intervention implementation	PLA groups—Stage 1: reflection	4 groups	8 groups
	PLA groups—Stage 2: from reflection to action		
	PLA groups—Stage 3: implementation of initiatives		
	PLA groups—Stage 4: evaluation		
Evaluation	Cost–benefit analysis	TBD	TBD
	Photovoice		
	Baseline questionnaire		
	Endline questionnaire		
	Endline qualitative Interviews		

PLA, participatory, learning and action; TBD, To Be Determined.

Our study builds on a pilot feasibility study of this approach in Cundinamarca-Colombia with a group of forty forcibly displaced persons.^6^

#### Phase 1: diagnostics (month 3–14)

The aim of this phase is to map out and understand community knowledge, the systems and services available at local level and everyday practices related to mental health. This is intended to identify the knowledge, practices, and resources available in the community and the experiences and beliefs held by community actors about mental health, mental illness and practices of care. Data collection initiated in April 2021 and was completed April 2022 for stages 1 and 2. Stage 3 remains ongoing. Specific aims, and procedures linked to this stage are as follows:

(*1) Assess local mental health systems capacities and capabilities in collaboration with service actors*. This stage involves three modes of data collection and engagement. First a review of existing mental health national interventions and their implementation and a Systematic Applied Policy Review of mental health national plans and policies currently in force. Second, involves motivated ethnographies^22^ of local mental health services and community needs, with semistructured interviews with service providers in each site. Third, includes focus groups with service providers, which are conducted online during the pandemic period. WhatsApp discussion groups are used as a platform to engage time-strapped institutional (psychologists, social workers) and community practitioners (including traditional healers) in both sites.^23^ The implementation of these steps is currently ongoing, having started in February 2021.

(*2) Explore community understandings of mental health, mental distress and well-being strategies in one urban and one rural PDET territory*. This involves a qualitative investigation of local understandings drawing on focus groups discussions, word association tasks, a Tree of Life exercise which focuses on experiences and community resources linked to achieving good mental health and well-being. It will also draw from the motivated ethnography in each site. Twelve focus groups discussions divided by gender and age are envisaged.

(*3) Work with local communities to evaluate appropriateness of standard mental health measures, using participatory methodologies*. Three standardised Mental Health measures PHQ-9 (Patient Health Questionnaire); WHO-5 (World Health Organisation- Five Well-Being Index), and Warwick-Edinburgh well-being scale were selected as potential screening tools to evaluate the impact of community designed activities. Initial team discussions with non-academic partners established the potential local appropriateness of the measures before they were discussed with community members. All measures have been standardised for use with Colombian or Spanish speaking participants.^24–26^ Focus groups will provide an opportunity to complete group cognitive interviews to explore meaning and perceptions of measures.^27^ This critical stage is informed by previous pilot work conducted in Colombia by members of our team.^6 28^

(*4) Assess the cost of the standard mental health services basket offer of local health systems*. The scarcity of data in these areas will make this stage challenging, but we are envisaging the potential collection of data from three sources: motivated ethnography, document analysis and service provider interviews (n – 30). This will allow us to understand comparative costing for community-led supports where possible.

#### Phase 2: intervention: PLA cycles to improve mental health community services (months 15–27)

The aim of this phase is to design and implement a community-led group intervention to (1) identify social drivers of mental health and priority conditions, (2) create shared spaces for dialogue and understanding of mental health, mental distress and well-being, identifying facilitators and barriers to collaborative processes of communication and action; and (3) establish priorities for action that improve community’s access to mental health services in PDET territories.

##### Intervention design

The intervention design is grounded in a participatory Theory of Change (TOC) process. Its first component is a participatory TOC workshop to involve large numbers of community members in the intervention co-design process. Participants from each community with interest in the project and their children were invited to a day long workshop in Florencia.

Drawing on preliminary analysis from the diagnostic phase, participatory activities are designed to facilitate real-time contributions to three main dimensions of the TOC process: *identification of challenges, assumptions, and preconditions, short and long-term outcomes and impacts*, and *backward chaining*. Manual development was led by RAB and refined by the academic team members. The TOC workshop manual is available in online supplemental materials, in English and Spanish. A summary of this process is provided in table 2.

10.1136/bmjopen-2022-069329.supp1 Supplementary data

**Table 2:** Theory Of Change (TOC) workshop structure

TOC session	Stage	Connection to TOC process	Activity to be conducted	Time allowance for activity	Number of facilitators required	Resources required
Session 1	Challenges that hinder good mental health and mental health services	Identify challenges, assumptions and context	Building problem trees	2 hours	2–4	Tape recorder Flip chart Paper Coloured marker pens Flash cards with themes from FGDs (5 full sets)
Session 2	Ideal world that enables good mental health and mental health services	Identify long-term outputs, other outputs and pathways to change.	Storytelling of an ideal world	1.5 hours	2–4	Tape recorder Flip chart Paper Coloured marker pens Photocopy of exercise
Session 3	Identify interventions which could be used to improve mental health and mental health services	Identify intervention and additional contexts.	Mapping and intervention building	1 hour	2–4	Tape recorder Cardboards Paper Coloured marker pens Flashcards

The TOC workshop was run in December 2021 facilitated by senior project members community researchers. A total of 44 people attended, equally split between each study site. Fourteen of these participants also attended the FGDs in phase 1. The sessions were audio recorded and data were transcribed and analysed in Spanish. The academic members of the project team used these data alongside preliminary analyses of focus group data and the focused ethnography, to develop a working model of the TOC. This was presented to the wider project team and community researchers, for evaluation and validation.

Based on the findings of the TOC process (see online supplemental data for final TOC), we identified that a participatory, learning and action (PLA) approach to the intervention would be an ideal structure. PLA cycles have been used widely in other resource-limited settings but to the best of our knowledge, our study is the first to implement PLA cycles at scale for community mental health improvement in Colombia. For example, their use has contributed to improved health outcomes for diabetes in Bangladesh,^29^ and maternal and child health in India,^30^ and are currently being evaluated for improvement in under-5 pneumonia in Nigeria.^31^ Crucially, our adaptation seeks to enhance links across groups that are historically opposed and limited by unequal access to power: community service providers, ex-combatants, internally displaced people and host community members. The value of these types of linking interventions for health systems improvement is well documented elsewhere.^32^

10.1136/bmjopen-2022-069329.supp2 Supplementary data

Based on community priorities identified in the TOC process, the proposed outcomes for the PLA intervention are as follows. We organise these into primary outcomes which we feel may be achieved in the short term, as well as longer term outcomes that could occur with longer running of PLA groups:

##### Primary outcomes

(1) Increased access to mental health acknowledge and information by community members; (2) improved feelings of belongingness and community cohesion and (3) improved perceptions of communication and relationships between practitioners and communities.

##### Long term outcomes

(1) Improved recognition of the importance of good mental health to wider health and well-being, (2) reduction of stigma around mental illness and mental health, (3) young people’s increased participation and communication in family life and community activities, (4) improved mental well-being, (5) improved experience of services (Respect, listening, communication).

##### Intervention structure

**Figure 1:**
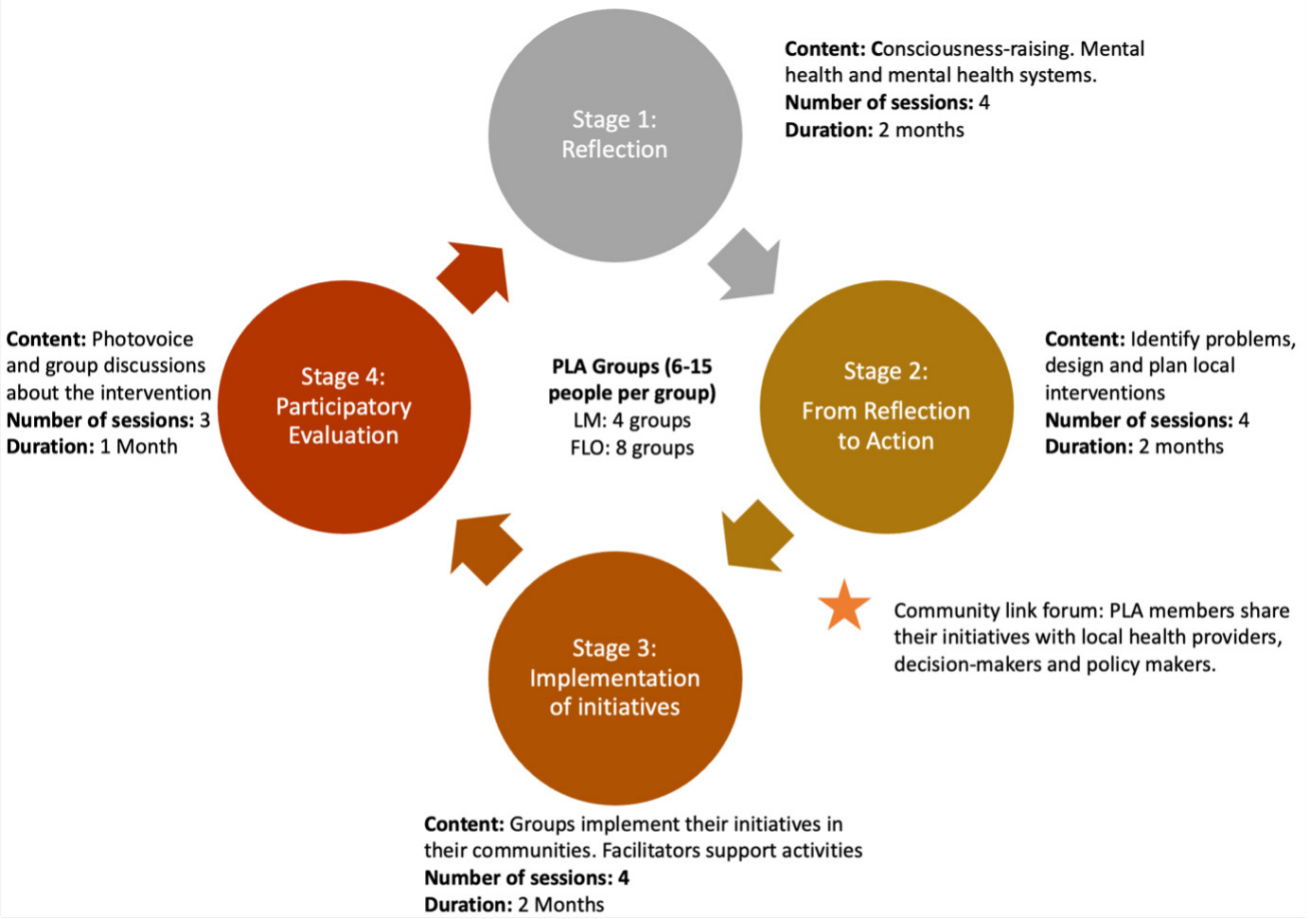
Participatory, learning and action (PLA) intervention structure.

The PLA intervention itself comprises 4 stages, running across 13 sessions (figure 1).

*Stage 1— knowledge building* is designed to provide community members with opportunities to develop new knowledge and understanding about mental health linked to the priority issues identified in the ToC workshop.

*Stage 2—from reflection to action* where participants will engage in a series of prioritisation and planning activities to identify a single challenge or focus and a plan for local action to address the issue. This stage will end with a community forum which creates a formal link between key actors in the local mental health infrastructure. Key actors were identified in the ToC and the ethnography and will be invited to engage in the community forums.

*Stage 3—implementation* will focus on groups’ implementation of their projects, and group led monitoring of the implementation process and the delivery of the planned activities. We will suggest the use of photography and video to help increase the accessibility of this process to community members.

*Stage 4—evaluation* will include a formalised participatory evaluation of each PLA group’s intervention, exploring any potential impact and efficacy in attaining the desired outcomes. Group members will be invited to participate in a photovoice project to achieve this. Phase 4 will also involve the election of community mental health champions. These individuals will become the focal points about mental health issues in their communities, combining with existing local infrastructure (such as health committees) in the long term. They will complete additional training provided by the project (ie, WHO quality rights training, Community MH gap training), as well as training on facilitating future cycles of the group for those projects who which to continue (see figure 1).

#### PLA group implementation

##### Group facilitator training

Community researchers are also facilitators for PLA groups. They completed full day of training, delivered in five short modules. The first of which included basic information about the project and the use of the manual. The next four modules correspond to each PLA phase outlining the objectives of each session and activities. To compensate for the short time period, the training programme was organised around role play activities, where facilitators completed all activities to be used within the intervention. Training also included a refresher on the processes for referrals (the same as used in phase one), and introduction to new data monitoring processes.

##### PLA groups development

Sessions will be delivered in a byweekly schedule, aiming to approximate two 3-hour sessions per month, running for 6 months to complete one cycle. Delivery of sessions will be supported by regular supervision by a member of the research team, as well as biweekly meetings with all community researchers, where implementation issues will be discussed. Due to time constraints created by the pandemic and funding instability created by geopolitical contexts in the UK, the pilot study will be restricted to a single cycle.

Group intervention structure will be determined by relevance to local context. In La Montañita, given the close ties between community members, it is likely that men and women will work together in groups in some cases. In Florencia, groups will likely be divided by sex and in both contexts will be divided by age, with young people meeting separately.

#### Phase 3: evaluation (months 20–27)

At programme level, we will explore the acceptability, appropriateness and feasibility of a PAR approach to establish platforms for community-led mental health systems strengthening. To evaluate this, we will hold monthly team meetings to discuss process and implementation challenges. We will also convene two workshops to discuss the strengths and weaknesses of the overall PAR approach and PLA intervention with team members and invited service delivery and community member representatives.

At the intervention level, we will explore standard process and outcome evaluation parameters as summarised in table 3, in line with MRC Complex intervention guidelines. For our intervention, we will evaluate potential impact at the individual and community level, combining traditional academic evaluations of outcomes using standardised measures, exit qualitative interviews with 30 participants (15 per site), and community-led evaluation methods—using photovoice methods.

**Table 3:** Outcome evaluation parameters for participatory, learning and action (PLA) group intervention

Item	Definition	Indicators	Target group	Frequency of collection	Person responsible	Source of data	Tool required	Data type
Acceptability	Satisfaction with the content and delivery of components	Experiences of sessions	PLA participants	Once	Research team	Midline/Endline interview	Topic guide	Qualitative
Appropriateness	Usefulness, relevance, suitability of component	Describing the intervention as useful	PLA participants	Once	Research team	Endline interview	Topic guide and survey	Qualitative and quantitative
						Endline questionnaire		
Feasibility	Suitability of component for routine implementation	Delivery of sessions	Community researchers	Once	Research team	Endline interview with community researchers	Topic guide and field diaries	Mixed
						Field diaries		
Fidelity of delivery	Delivery of the component as intended	Number of sessions conducted	PLA participants	Once	Community researchers	Attendance registers	Attendance registers	Quantitative
		Content of sessions	Community researchers	Monthly	Community researchers	Field diaries	Field diaries	Mixed
		Participatory-ness of the sessions		Monthly	Community researchers	Field diaries	Field diaries	Qualitative
Fidelity of receipt	Intervention reach	Number of attendees	PLA participants	Weekly	Community researchers	Attendance registers	Attendance registers	Quantitative
		Profile of participants	PLA participants	Once	Community researchers	Questionnaire	Questionnaire (demographic session)	Quantitative
	User understandings and performance resulting from receipt of component	Community-led intervention strategies	PLA participants	Once	Research team	Field diaries and endline interviews	Topic guide and field diaries	Mixed
		Photovoice activities	PLA participants	Once	Community researchers	Photovoice	FG discussions and images	Qualitative

At the individual level, we will measure impact using standardised measures tested and validated by the community in phase 1. These measures are summarised in table 4. Where standardised tools were not available, we developed specific items to explore dimensions of knowledge, behaviour and practices linked to mental health. This was informed by knowledge attitudes and practices (KAP) studies in other areas^33^ and a similar tool used by other large scale mental health studies.^34^ To better understand community and systems-level impacts, we will also run simulations to assess the cost–benefit or the cost-effectiveness of the actions that are (1) implemented and (2) planned in phase 2. When it makes sense to monetise and data are available, results will be monetised using current knowledge of different uses of time by young individuals (education, work, political engagement, working for their communities) in resource-constrained countries for the cost–benefit analysis. When not possible, cost-effectiveness analysis will be developed. Costs will be estimated using the baseline quantification of cost of health services in WP1, if possible. Together, these strategies evaluate the pathways, mechanisms and resources required for promoting and improving mental health services and inform future questions to be considered in future trials and scaling up of our intervention.

**Table 4:** PLA intervention—outcome evaluation measures

Long term outcomes	Indicator	Measure
Improved experiences of mental health reduced symptoms of mental ill health	Improved well-being Reduced symptoms of depression	WHO-5 (5 items)
		PHQ-2 (2 items)
Short-term outcomes	Indicator	Measure
Improved perceptions of quality of relationships between practitioners and communities	Increased willingness to seek treatment	Perceptions on different Service providers (5 items)
Improved feelings of belongingness and community cohesion	Increased sense of attachment to place/home	Sense of belonging and attachment to place^40^ (14 items)
	Increased feelings of emotional and community support Increased feelings of inclusion and acknowledgement in the community	World Bank Social Capital measure (17 items)
	Improved perception of individual and collective agency Positive sense of self/identity	Possible selves questionnaire^41^ (6 items)
Increased mental health literacy knowledge attitudes and practices questions	Increased mental health literacy	Depression symptom knowledge (5 items)
		Stress symptom knowledge (5 items)
		Substance misuse symptom knowledge (5 items)
	Greater acceptance of others seeking treatment	3 items
	Helping others to seek treatment	2 items
	More positive perceptions of mental illness	1 item
Reduction of mental health stigma	More willingness to discuss/explore mental health needs in communities and families	RIBS reported behaviours subscale (4 items)

### Sampling

Across the project two sampling strategies were used. For the diagnostic phase, purposive sampling ensured selection on the basis of participants’ characteristics^35^ in our case, in-depth knowledge of the context and local mental health services, from both potential service users’ and providers’ perspectives. Within this framework, we adopted a maximum variation approach, selecting across a broad spectrum of characteristics which included age, gender and mental health status. This will support an in-depth understanding of the range of different groups who populate PDET communities ensuring saturation of contexts, through triangulation of data and experiences.^36^

Inclusion and exclusion criteria will be uniform across the programme. Inclusion criteria for community members will include (1) place of residency (Florencia/La Montañita), reported by the participant as their home; (2) age (16–25 years old and 26+years); (3) willingness to voluntarily participate (inform consent signed) and (4) self-reported emotional distress experiences. Service provider sampling will include (1), working in a health provider setting or in a decision-making scenario related to the health field will be used in addition to the criteria used for community members as an inclusion criterion. Those with untreated mental health affections, people unable to give consent, people under 16 years old, and people unrelated to health providing systems and institution in the case of health representatives will not be eligible for participation in our study.

For the intervention, purposive sampling will be used to include community members who participated in the diagnostic phase as well as availability sampling to include a wide range of other community members. We did not conduct a formal sample size calculation due to the lack of data on the expected intervention effect size linked to our outcomes. However, simple power analyses linked to the use of scales such as the PHQ-9 indicate that a sample size of approximately 30 is required to show significance changes in pre–post testing. Notwithstanding, our recruitment aims were guided by previous experience of the research team applying this method in similar populations in Colombia^6^ where the attrition rate was found to be around 42% among a similarly highly mobile and critical population. This is similar to other projects working with vulnerable and transient populations in PDET territories in Colombia (Idrobo *et al*, personal communication).

### Data analysis

Qualitative data across all phases will be analysed using thematic network,^37^ reflexive,^38^ or framework analysis.^39^ Thematic network analysis will be used to understand community perceptions of well-being and emotional distress, and local mental health services. Other thematic analysis methods mentioned will be used for analysing data derived from the motivated ethnography, qualitative data from our evaluation, and in the policy review to identify primary topics regarding access and mental health services in Colombia, particularly in PDET municipalities. Collaborative data analysis strategies will be applied across all our project analysis, involving participants and community researchers in data analysis, verifying outputs and guaranteeing data validity.

Descriptive analysis and simple regression modelling will be performed on quantitative data from our evaluation questionnaire to evidence changes regarding mental health and well-being, and community-level outcomes (social capital and social belonging) before and after our intervention. These changes will be captured comparing baseline and endline results following the completion of the intervention.

### Patient and public involvement

Because of the nature of PAR research and our overall coproduction approach, this project is committed to public involvement. Community partner organisations were involved in the framing and development of the project from the outset (including funding application stages) and are involved in major planning and decision-making. Intervention design processes involve everyday citizens, or ‘potential service users’ during all phases. The TOC approach planned for this study is rooted in participant and public involvement, diverging from other approaches that involve a handful of patient representatives, or make use of previously collected data from wider communities. Instead, the stage will include people with previous experience of mental health services, family members, friends and potential service users within the TOC process.

### Ethics and dissemination

Ethical approval has been obtained from two academic institutions. One in Colombia (2021–1393) and the UK (16127/005). We will disseminate our work across academic, policy and community platforms. We will produce peer-reviewed publications and policy reports, alongside public communication activities such as workshops, short-films, infographics and photography exhibitions to highlight community projects. A detailed communication strategy will be finalised based on collaborative agreement across our entire team and policy stakeholders.

## Supplementary Material

Reviewer comments

Author's manuscript
