# Supplementary material for: Using participatory action research to reimagine community mental health services in Colombia: a mixed-method study protocol
Source: BMJ Open. 2022 Dec 21;12(12):e069329. doi: 10.1136/bmjopen-2022-069329 (PMC9772630; doi:10.1136/bmjopen-2022-069329)
Supplement: Supplementary data [file bmjopen-2022-069329supp001.pdf]

Starting From the Bottom: Building a Theory of Change (ToC) for community interventions to improve mental health services in PDET communities in Colombia

STARS-C project

Manual
English Version

Copyright © STARS-C project. Please contact project PI Dr. Rochelle Burgess prior to reproduction/reuse

Theory of Change Workshop Manual

Methodology:

Conduct a public community forum and a Theory of Change workshop to collectively develop expectations, priorities and desired outcomes of mental health and mental health services for communities. This will also create an opportunity to set a broader goal for what people would like to see as the main outcomes of participation in through in this project.

Sampling:

50 participants

Procedure:

The below table provides a summary of what will be done in each session, and what the aim of each session is.

TOC session	Stage	Activity to be conducted	Time allowance for activity	# of facilitators required	Resources required
**Session 1**	Challenges that hinder good mental health and mental health services	Building problem trees	2 hours	2-4	1. Tape recorder<br>2. Flip chart<br>3. Paper<br>4. Coloured markerpens<br>5. Flash cards with themes from FGDs (5 full sets)
**Session 2**	Ideal world that enables good mental health and mental health services	Storytelling of an ideal world	3 hours	2-4	1. Tape recorder<br>2. Flip chart<br>3. Paper<br>4. Coloured markerpens<br>5. Photocopy of exercise
**Session 3**	Identify interventions which could be used to improve mental health and mental health services	Mapping and intervention building	1 hour	2-4	1. Tape recorder<br>2. Cardboards<br>3. Paper<br>4. Coloured markerpens<br>5. Flashcards

Copyright © STARS-C project. Please contact project PI Dr. Rochelle Burgess prior to reproduction/reuse

Things to remember:

1. **Each session should be audio recorded to be transcribed/translated later.**
2. **You must make sure you take photos of all the outputs from each activity (e.g. problem tree etc).**

Introduction

We provided information about the project and the team, for participants to feel welcome and know who to ask if any questions should arise.
With the help of attendees, we developed a set of rules for respectful groups discussions and maintaining confidentiality.
Each participant was given a name tag, assigned a group number, and was sat on a table with the rest of their group. Facilitators prompted them to introduce themselves while activities started, as they would be working together throughout the day.

Session 1
Where we begin: Mapping and connecting factors that shape poor mental health

The aim of this session is to identify challenges that hinder good mental health and mental health services. We will do this, through using flash cards, which summarise the findings from our earlier focus group discussions, to build problem trees. When summarising the focus group discussions' data, be sure to avoid interpretations. The summary should be as much as possible a descriptive summary of raw data.

Step 1. *Brief introduction to the topic:* Remind participants of the activities during the FGDs and discuss the themes that emerged. You may want to facilitate a brief discussion to help warm up the room. For example, each facilitator is given a stack of randomized flash cards to distribute across the room. Then ask participants to place them into 'categories' on the walls.

Step 2. Divide participants into smaller groups. The groups should reflect the way that we will organize the PLA groups. Each group should have no more than 10 people.

Step 3: Assign the following topics to each group for them to create a problem tree.

1) Group of adults A (Florencia) – Mental health
2) Group of adults B (Florencia) – Mental health services
3) Group of young people (Florencia) – Mental Health
4) Group of adults A(La Montañita) – Mental health services
5) Group of adults B(La Montañita) – Mental health
6) Group of young people (La Montañita) – Mental health services

Step 4. Introduce the main activity – the problem tree (below) and provide instructions as follows:

Script: Today, we want to think deeply about the challenges that hinder good mental health and mental health services. We can articulate problems very clearly,

Copyright © STARS-C project. Please contact project PI Dr. Rochelle Burgess prior to reproduction/reuse

but this task will help us to build connections between challenges at various levels in our lives. We can think of this more clearly, if we think about something physical in our environment, like a tree. A tree has different parts that all connect to make the whole. The roots, which are hidden, not always visible, but make it possible for the tree to exist. They grow first and have the largest effect. The main part of the tree – which is the trunk. It connects the roots to the outside world – it is the part that we see first, that is most visible. Finally, the leaves – the top of the tree, they grow up and out into the future.

The activity we will do first, is to build a problem tree, which helps us to make sense of these major themes that emerged from our focus group discussions. In your groups, you need to think about yourselves – as women, men, young people, and what specific problems matter the most to you, in your lives, and connect them from the ‘roots’ to the broader outcomes.

Each problem tree is split into three sections: the root (foundations/root causes) the core problem (what we can see) and the outcomes/consequences.

The roots are where you may map the root/hidden causes of challenges, such as unemployment, weak relationships; conflict; violence. The trunk signifies what the main problem is. For some people, this could be a mental health condition (depression), but it could be many other things as well (no education; isolation; hunger; family separation). Finally, at the branches, this signifies the outcomes, or the consequences of these difficulties. This could include things like: loss of work; low self-esteem; mental health challenges; exclusion, etc.

NB to facilitator: it may help, to build an example tree, while you are discussing these points above. You should have example flash cards to put in each part of the tree and ask participants where to put each.

Using the cards you have as a starting point, begin to build your problem trees. Some groups will make a tree for the experience of poor mental health, and the others will make a tree for what hinders mental health services. You will also be given blank cards, if there are things that were not captured in prior focus groups, but that you think are important to consider.

If it helps, you can imagine a person that you know, or that you have heard of, who is living through these issues right now. How would you build a tree to describe their life and experiences? How would you build a tree to describe their quest to seek treatment/support with the things they find difficult?

Instructions: Hand out cards to each group, showing the themes that emerged during the FGDs. Show participants the example problem tree below and give them 1 hour to discuss and create problem trees within their groups. In each group, provide a recorder device to capture the discussions being held by the participants.

Copyright © STARS-C project. Please contact project PI Dr. Rochelle Burgess prior to reproduction/reuse

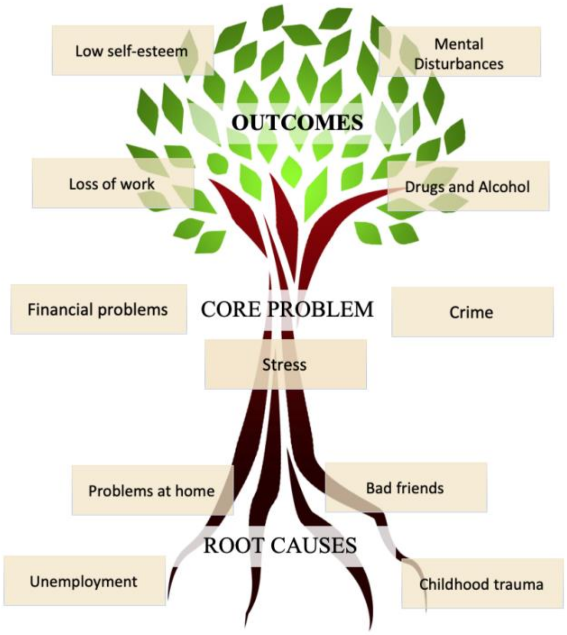

Step 5. After 1 hour, ask a representative from each group to share their problem tree with the rest of the participants (which should take approximately another hour).

Step 6. While the participants share their trees, one facilitator should be taking notes to support later analysis. Another should be taking more general notes to facilitate discussion. Note the similarities and differences between trees, and the challenges and outcomes of healthcare vs health services. These should be shared with the wider group, and participants should be asked for their thoughts on what is being shared.

Session 2
Storytelling of an ideal world – imagining outcomes and outputs

The aim of this session is to identify potential solutions to improve mental health services, and mental health outcomes. This is a long-term plan but should give participants a chance to think about what actions are required to achieve this long-term vision.

Step 1. Facilitators present the following phrase.

"The way we think about the future often focuses on the immediate future. However, when thinking is inspired by a vision, there is more room to achieve things which are thought of as 'unthinkable'. A vision for a better future gives us hope and increases motivation to take action to pursue that vision"

Copyright © STARS-C project. Please contact project PI Dr. Rochelle Burgess prior to reproduction/reuse

Step 2. Participants should work in the same groups from activity 1.

Script: "Imagine your community 20 years from now. The national television agency (Día a Día/Séptimo Día) has prepared a programme on the outstanding achievements your community has made to increase the rates of access to mental health services and improving mental health in the community. The television/radio programme was prepared based on interviews with community members, local authorities, traditional leaders, and health institutions working in the district. Imagine what the programme would report about your community's achievements in mental health. They have completed a special feature, on two people who have experienced this change. One person is someone whose mental health has been improved, and another is a practitioner who has worked with the patient and the community to build that change.

NB for facilitators: These questions should be handed out to each group on a piece of paper

General questions to consider for all parties

- ❑ What are major changes your community has made in the last 20 years to ensure good mental health in your community?
- ❑ What are the major changes your community has made in the last 20 years to increase the rates of access to mental health services?
- ❑ As a mental health provider, ¿what have you done to improve the mental health of your community?
    - Example: If you are a psychologist, how did you help your community?
- ❑ How have community leaders have supported efforts to address poor mental health?

Questions for your main characters:

- ❑ What actions did you do to start making life changes in terms of your mental health? Who was involved?
- ❑ What action plan did they follow in the first year to make the change happen?
- ❑ How did they convince other people who are important in their lives that this was the right decision?
- ❑ How did they keep going in the long run?

Scrip continued: "in your groups, you will need to write a story about this future world. It may help you to think about the questions in on the attached sheet of paper. You will present your story to the group in a role play (no more than 10 minutes long) of a television interview. There should be four speaking roles:

1) The journalist (who could be asking some of the questions we have provided)
2) a main character who has benefitted from the new world and services (could be the same person you thought about to help you do activity 1)
3) a health care provider
4) A key person who you feel is important to the story. (i.e could be a family member, a community leader, a politician, a friend, etc)

You will have 1 hour to work on this.

Copyright © STARS-C project. Please contact project PI Dr. Rochelle Burgess prior to reproduction/reuse

Step **3**. After 1 hour, ask the groups to present their plays within each site. Then ask them to vote for the better story as this will be presented to the broader group including participants from the other site. After deciding which play to present, ask participants to add or improve their stories if they think they should.

Step 4. Finally, let participants present the play from each site to one another. The facilitators should be taking notes and asking people to think about similarities or make comments towards what is being presented after each play. Audio and video record each presentation and the plenary discussion for future analysis.

Session 3
Mapping and intervention building

The aim of this final session is to identify interventions which could be used to improve better mental health and mental health services in communities. It may be worth stating at this stage, that these discussions will shape how we run the second stage of our project – which are the activities we facilitate to improve mental health and improve relationships between mental health services and communities over the course of the next year.

Step 1. Divide participants into same groups as for previous activity

Step 2. Explain that they will need to think back to the problem trees from Activity 1 and what was discussed in Activity 2.

Step 3. Tell participants that they have 1 hour to consider these challenges and imagine possible solutions. Make cardboards with the following questions.

1. What are the interventions you need to improve mental health and mental health services in your community?
2. What resources do you need to implement those interventions/actions?
3. What are the expected outcomes of implementing those interventions?

Step 4: Ask participants to write down the answer to those questions in flashcards and then to paste them under each cardboard.

NB to facilitator: Register the answer provided. If there is enough time, share results with the broader group trying to highlight similarities and differences.

Closing statements

Participants were thanked for their time and contributions. Facilitators went around the room asking about people's experiences and any feedback for future activities.
Facilitators shared next-steps for the project to have a sense of continuity and stay in touch with the community.

Copyright © STARS-C project. Please contact project PI Dr. Rochelle Burgess prior to reproduction/reuse
