# Supplementary figures and images for: Using participatory action research to reimagine community mental health services in Colombia: a mixed-method study protocol
Source: BMJ Open. 2022 Dec 21;12(12):e069329. doi: 10.1136/bmjopen-2022-069329 (PMC9772630; doi:10.1136/bmjopen-2022-069329)

ToC STARS-C Project

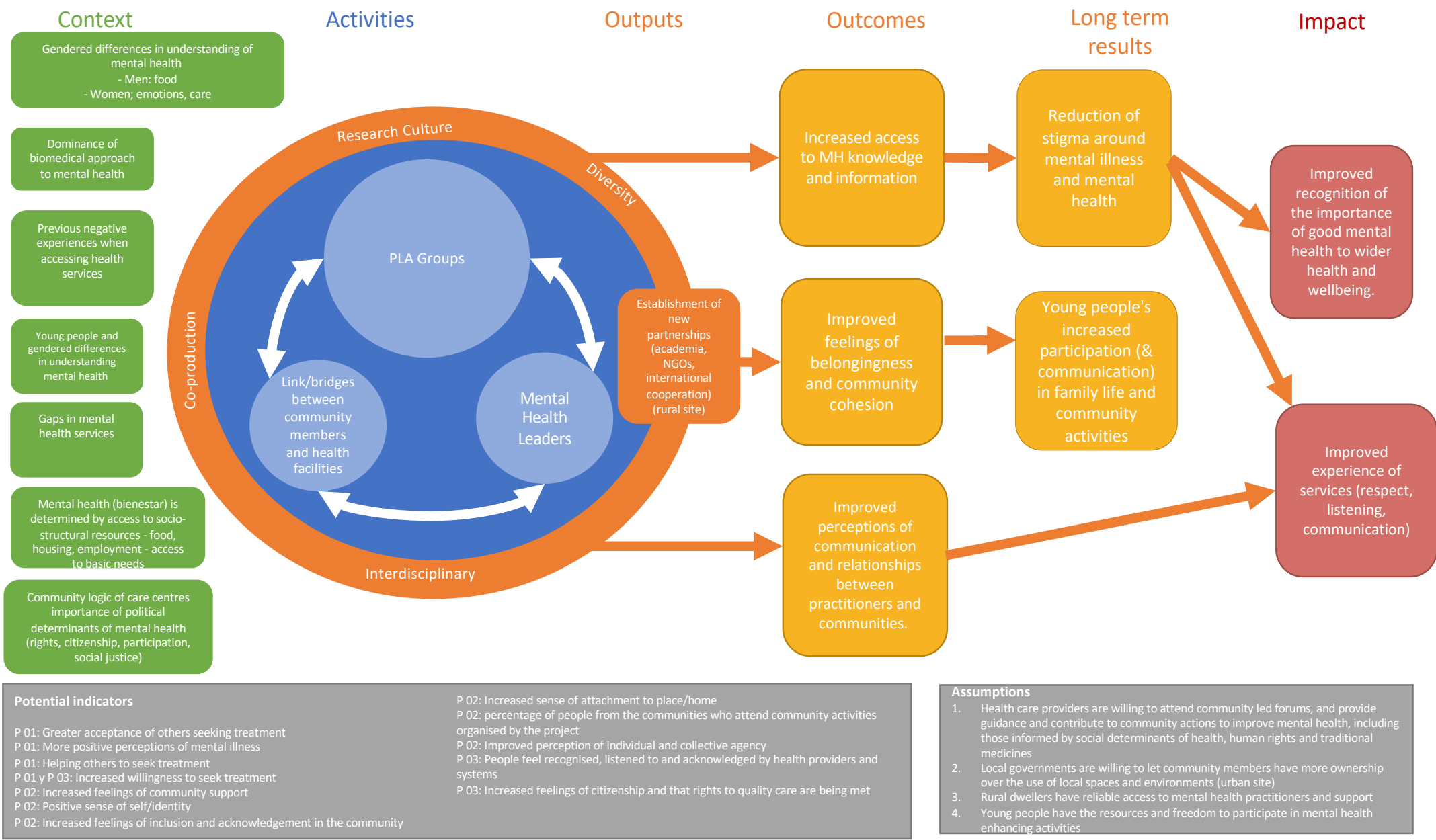

Supplement: Supplementary data [file bmjopen-2022-069329supp002.pdf]
