# Supplementary material for: Using participatory action research to reimagine community mental health services in Colombia: a mixed-method study protocol
Source: BMJ Open. 2022 Dec 21;12(12):e069329. doi: 10.1136/bmjopen-2022-069329 (PMC9772630; doi:10.1136/bmjopen-2022-069329)
Supplement: Author's manuscript [file bmjopen-2022-069329.draft_revisions.pdf]

BMJ Open is committed to open peer review. As part of this commitment we make the peer review history of every article we publish publicly available.

When an article is published we post the peer reviewers' comments and the authors' responses online. We also post the versions of the paper that were used during peer review. These are the versions that the peer review comments apply to.

The versions of the paper that follow are the versions that were submitted during the peer review process. They are not the versions of record or the final published versions. They should not be cited or distributed as the published version of this manuscript.

BMJ Open is an open access journal and the full, final, typeset and author-corrected version of record of the manuscript is available on our site with no access controls, subscription charges or pay-per-view fees (http://bmjopen.bmj.com).

If you have any questions on BMJ Open's open peer review process please email info.bmjopen@bmj.com

BMJ Open

Using Participatory Action Research to re-imagine community mental health services in Colombia: A Pilot Study Protocol

Journal:	*BMJ Open*
Manuscript ID	bmjopen-2022-069329
Article Type:	Protocol
Date Submitted by the Author:	18-Oct-2022
Complete List of Authors:	Burgess, Rochelle; UCL Institute for Global Health<br>Dedios Sanguineti, María Cecilia; Universidad de Los Andes, School of Goernment<br>Maldonado-Carrizosa, Darío; Universidad de los Andes, School of Government<br>Fonseca, Laura; London School of Economics and Political Science, Department of Psychological and Behavioural Science<br>Vera San Juan, Norha; UCL Institute for Global Health; University College London, Rapid Research Appraisal and Evaluation Lab (RREAL)<br>Lucumí, Diego; Universidad de Los Andes, School of Government<br>González-Gort, Mónica; Universidad de Los Andes, School of Government<br>Melgar, Mónica Carreño; Universidad de los Andes, School of Government<br>Gaviria, María Fanny; Corporación Manigua<br>Tovar, Diego; Cooperativa multiactiva para el buen vivir y la paz del Caquetá<br>Jovchelovitch, Sandra; London School of Economics and Political Science, Department of Psychological and Behavioural Science
Keywords:	MENTAL HEALTH, Depression & mood disorders < PSYCHIATRY, PUBLIC HEALTH

SCHOLARONE™
Manuscripts

Using Participatory Action Research to re-imagine community mental health services in Colombia: Pilot Study Protocol

Corresponding author:

Rochelle A. Burgess,
Institute for Global Health,
30 Guilford Street
Rm. 3.04,
London
WC1N 1EH
r.burgess@ucl.ac.uk.

List of authors

Rochelle A. Burgess, Institute for Global Health, University College London, London, United Kingdom.
María Cecilia Dedios Sanguineti, School of Government, Universidad de Los Andes, Bogotá, Colombia.
Darío Maldonado-Carrizosa, School of Government, Universidad de Los Andes, Bogotá, Colombia.
Laura Fonseca, Department of Psychological and Behavioural Science, London School of Economics and Political Science, London, United Kingdom.
Norha Vera San Juan, Institute for Global Health, University College London, London, United Kingdom.
Diego Lucumí, School of Government, Universidad de Los Andes. Bogotá, Colombia.
Mónica González-Gort, School of Government, Universidad de Los Andes. Bogotá, Colombia.
Mónica Carreño Melgar, School of Government, Universidad de Los Andes, Bogotá, Colombia.
María Fanny Gaviria, Corporación Manigua (CORPOMANIGUA), Florencia, Colombia.
Diego Ferney Tovar, Cooperativa multiactiva para el buen vivir y la paz del Caquetá (COOMBUVIPAC), La Montañita, Colombia.
Sandra Jovchelovitch, Department of Psychological and Behavioural Science, London School of Economics and Political Science, London, United Kingdom.

Word count: 4,189

For peer review only

Key words

Colombia; mental health; health systems strengthening; community participation; community involvement

Authors and Institutions

Rochelle A. Burgess, r.burgess@ucl.ac.uk, University College London.
María Cecilia Dedios Sanguineti, m.dedios@uniandes.edu.co, Universidad de Los Andes.
Darío Maldonado-Carrizosa, dmaldonadoc@uniandes.edu.co, Universidad de Los Andes.
Laura Fonseca, l.m.fonseca-duran@lse.ac.uk, London School of Economics and Political Science.
Norha Vera San Juan, n.verasanjuan@ucl.ac.uk, University College London.
Diego Lucumí, di.lucumi@uniandes.edu.co, Universidad de Los Andes.
Mónica González-Gort, m.gonzalez22@uniandes.edu.co, Universidad de Los Andes.
Mónica Carreño Melgar, m.carrenom@uniandes.edu.co, Universidad de Los Andes.
María Fanny Gaviria, f.gaviria@corpomanigua.org, Corporación Manigua (CORPOMANIGUA).
Diego Ferney Tovar, pacercri@gmail.com, Cooperativa multiactiva para el buen vivir y la paz del Caquetá (COOMBUVIPAC).
Sandra Jovchelovitch, s.jovchelovitch@lse.ac.uk, London School of Economics and Political Science.

ORCID Ids

Rochelle A Burgess: 0000-0001-9749-7065
María Cecilia Dedios-Sanguineti: 0000-0002-6141-304X
Darío Maldonado-Carrizosa: 0000-0003-3544-208X
Laura Fonseca: 0000-0003-0638-3447
Norha Vera San Juan: 0000-0002-8677-7341
Diego Lucumí: 0000-0003-1834-7937
Mónica González-Gort: 0000-0002-5488-832X
Mónica Carreño Melgar: 0000-0003-2579-8568
María Fanny Gaviria: N/A (Non-academic partner)
Diego Ferney Tovar: N/A (Non-academic partner)
Sandra Jovchelovitch: 0000-0002-0073-2792

For peer review only

ABSTRACT

Introduction

Mental-health care systems are challenged by how they hear and respond to what marginalised communities experience as drivers of mental distress. In Colombia, this challenge intersects with wider challenges facing post-conflict reconstruction. Our pilot study will explore the feasibility and acceptability of a participatory approach to developing community-led participatory interventions for community mental health systems strengthening and mental health improvement, in two sites in Caquetá, Colombia.

Methods and analysis

The project is divided into three distinct phases aligned with community participatory action research (PAR) cycles diagnostic, intervention, and evaluation. This allows us to use a participatory approach to design a community-led, bottom-up community intervention for mental health systems strengthening and the promotion of mental health and wellbeing.

The diagnostic phase explores local understandings of mental health, mental distress, and access to mental health services from community members and health providers. The intervention stage will be guided by a participatory Theory of Change process. Community priorities led to the development of a Participatory, Learning and action (PLA) informed group intervention, with a community linkage forum. The pilot of the PLA intervention will be evaluated using MRC process evaluation guidelines.

Ethics and dissemination

This project has received ethical approval from two sources. Universidad de Los Andes [2021-1393] and the University College London [16127/005]. Dissemination of findings will include academic publications, community forums, policy briefs, and visual media (cartoons, pod casts and short films).

For peer review only

Article summary - Strengths and limitations of this study

- This pilot study aims to provide evidence for a new methodology that meaningfully involves citizens developing and strengthening health systems in complex settings.
- The study pilots for the first time in Colombia the use of participatory and learning action groups (PLA) for improving mental health and strengthening community mental health systems.
- Our approach will yield novel understandings and processes that enable better collaboration between community knowledge systems, community members, and the services that are designed to support them.
- The main challenge facing this pilot is the integration of participation across multiple sectors. Our desire to include potential service users and service providers in PAR processes will face difficulties in the pandemic environment and in a context where mental health services are limited.
- PAR processes can be directly impacted by wider geopolitical realities – such as the UK government funding cuts, which disrupted community processes and relationship building in the early stages of our work.

For peer review only

INTRODUCTION

Globally, the burden of mental health conditions is shaped by gaps in services. In Low-Middle Income Countries (LMICs), 75% of the population lacks access to any form of care.[1] The COVID-19 pandemic exacerbated these challenges as intersecting social realities deepen distress, increase the incidence of mental health disorders, and overburden health systems.[2] In the case of Colombia, political violence, poverty, and displacement further aggravate this burden. Previous research shows that victims of armed conflict are more likely to suffer from mental health disorders,[3] with poverty explaining 86% of mental health inequalities in the country.[4]

Six years after the Peace Accords between the Colombian Government and the FARC guerrilla, there are still barriers in the implementation of the Psychosocial Care and Comprehensive Health Services for Victims programme (PAPSIVI) and the Psychosocial Wellbeing Component in the reintegration route for ex-combatants (Resolution n. 4309). In the case of ex-combatants, a dual status of victims and perpetrators requires balancing psychosocial well-being, personal protection, and political acceptability of mental health services. This population, like the victims of the conflict, reside in rural areas where services are scarce or non-existent.[3,5]

Mental-health care systems are challenged by how they hear and respond to what marginalised communities experience as drivers of mental distress.[6–8] This is acknowledged by global,[9] and national priorities, which call for providing accessible and quality services to overlooked communities. In Colombia, this includes territories and rural populations (campesinos) that are the focus of Territorially Focused Development Programmes (PDETs in Spanish), a national programme of development prioritising those who have been heavily affected by disproportionate armed conflict, poverty, illicit economies, and institutional fragility.[10,11]

For peer review only

Scaling-up services is important but only a partial response; sustainable solutions to improve mental health require dialogue between health systems and communities.[12] Community-owned and anchored interventions are critical to re-establishing trust between local populations and systems, particularly after periods of extended upheaval. In this context, integrating community-level experiences of mental health and mental distress with institutional responses by state-level actors is a necessary step towards effective community mental health services. This requires a multi-level interdisciplinary perspective that links individual and community well-being to wider institutional, socio-economic, and political contexts. Community Participatory Action Research (CPAR) approaches allow us to explore the ability to identify strengths and solutions produced by communities for communities, connecting them to wider systems, while acknowledging them as agents with the capacity to create effective, context sensitive solutions.[13]

As Colombia begins to refocus its efforts towards achieving these global and national policy aims, three critical areas require attention: 1) Wider social and political contextual factors that drive experiences of poor mental health,[14] 2) Increasing understanding of local embodied knowledge and lived experiences of communities and their relevance for building knowledge about mental health,[15] and 3) The role and resources offered by community participation in the co-design of interventions and services that are effective.[8]

In response to these demands, we will implement a participatory process to design, implement, and evaluate a participatory intervention to strengthen community mental health care systems in two PDET communities in Caquetá-Colombia. We are guided by the following research question: *what are the pathways, mechanisms, and resources needed to catalyse collaborative action between communities and institutions for promoting and improving mental health services for PDET communities?* To this end, we aim:

For peer review only

(1) To co-design and co-implement a participatory group intervention to create trust and opportunities for collaborative action between community and health system actors to improve the performance of community mental health services.

(2) To co-evaluate the group intervention in terms of process, outcomes (including individual and community mental health), and simulations of the cost-benefit and cost-effectiveness of the intervention at individual, community, and health services levels.

(3) To produce a manual based on the development, implementation, and evaluation of the intervention to guide communities and institutions in the application of these methods for developing and scaling up community mental health services in Colombia. We expect these tools to be made widely applicable in other low-resource or conflict-affected settings.

The project is divided into distinct phases aligned with community participatory action research (PAR) cycles reflecting diagnostic, intervention and evaluation. This protocol presents the STARS-C objectives, procedures, and methodological considerations for implementing a participatory mental health research project in conflict areas amidst the Covid-19 pandemic.

METHODS AND ANALYSIS

The project will be implemented in inter-related phases aligned with participatory action research (PAR). It will run from February 2021 to May 2023 in Caquetá, Colombia. Implementation of the group intervention will run from July 2022 – March 2023. The project has been co-designed through existing partnerships involving academics and two community-based organisations: (1) the Manigua Corporation [*Corpomanigua*], an organization of women with experience in the design and implementation of projects with marginalized communities, located in Florencia, representing an urban community and (2) the Multi-active Cooperative for Wellbeing and Peace of Caquetá (*Cooperativa Multiactiva para el Buen Vivir y la Paz del*

For peer review only

Caquetá- (COOMBUVIPAC), which represents a rural community of ex-combatants from the former guerrilla FARC-EP, located in the small village (*vereda*) Héctor Ramírez Poblado Center (CP-HR - former Territorial Space for Training and Reincorporation Héctor Ramírez) in the municipality of La Montañita.

Co-design and co-implementation will be further achieved through the appointment of community researchers (two from each site), who live and work in the communities being studied, and are not previously employed by our NGO partners. They will be involved in all stages of the implementation of the project as detailed below and were appointed prior to the drafting of this protocol. To ensure more equal partnerships in this work community researchers were trained in collecting qualitative information, quantitative questionnaires and in psychological first aid to support potential psychological and emotional distress among participants. Regular supervision is provided in real-time planned meetings. WhatsApp groups are utilised for constant communication.

Setting

Caquetá is one of the 32 departments of Colombia, and the only region of the country in which all municipalities are included in the Territorially Focused Development Plans (PDET in Spanish). The project will be conducted in 2 of these PDET municipalities: Florencia and La Montañita. Each of the municipalities also represents diversity within a more general context of deprivation and adversity.

Florencia is Caquetá's capital city and constitutes its largest population with 173,011 inhabitants, [16]. Updated mental health statistics are not available at the municipality level; however, a report by MSF (2010) in Caquetá suggests that of the 60% of the nearly 5000 patients affected by armed conflict and internal displacement, 18% were diagnosed with adaptative disorders, 18% with relationship problems and problems associated with abuse or neglect , 11% by major depression with one episode, 9% with grief and 8% with mood

For peer review only

disorders [17]. Arguably, the prevalence of these mental health disorders relates to structural drivers such as high unemployment levels. According to the latest report done by the National Administrative Department of Statistics in 2020, the unemployment rate in Florencia was 25%, with women having a higher unemployment rate (29.2%) than men (21.5%),[16] both much more, than the current unemployment national rate of 11% [18]. As an urban area, Florencia has access to some specialized mental health facilities and staff, including psychologists, psychiatrists and nurses.

La Montañita is a rural area located to the south-west of Florencia and one of the areas most affected by the armed conflict, with 8,756 victims out of a total of 14,692 inhabitants[16,19]. No mental health statistics are available for the municipality but reports from local organisations point to mental distress associated with poverty and conflict as well unmet care needs. The project will be carried out in a small village (*vereda*) self-named *Centro Poblado Héctor Ramirez,* which is one of the former territorial spaces for training and reincorporation for former FARC-EP combatants (AETCR in Spanish) in La Montanita.

Design

The STARS-C programme outlines a three-phase process to guide stakeholders in the development and strengthening of community led mental health systems. It is informed by coproduction principles, to enable a platform for involving community members in a process of thinking through what changes are needed to improve access to, and the quality of mental health services[20]. Coproduction principles demand the inclusion of everyday actors, or potential service users, within processes of design and development. We will achieve this through involving everyday community members using Community Participatory Action Research (CPAR) [21] model, to thinking through what changes are needed to improve access to and quality of mental health services[20]. As such the project combines participatory

For peer review only

qualitative inquiry across its three phases of diagnosis, intervention, and evaluation (see table 1) with quantitative assessments of mental health outcomes in a process described below. Our study builds on a pilot feasibility study of this approach in Cundinamarca-Colombia with a group of forty forcibly displaced persons.[6]

Table 1.
Phases and data collection strategies

Phase	Data Collection	Participants	
		La Montañita	Florencia
Diagnostic	Focus Group 1: Local understandings of mental health and mental distress- Tree of Life	n=42	n=57
	Focus Group 2: Evaluation of standardised measures of mental health	n=34	n=49
	Interviews Health Providers	n= 13	n=17
	Whatsapp focus groups Health Providers	n=11	n=10
	Motivated Ethnography (1 month)	Local Hospital-Community health post	City Hospital
Intervention Design	Theory of Change Workshop	n=25	n=25
Intervention implementation	PLA Groups-Stage 1: Reflection	4 groups	8 groups
	PLA Groups-Stage 2: From Reflection to action		
	PLA Groups-Stage 3: Implementation of initiatives		
	PLA Groups-Stage 4: Evaluation		
Evaluation	Cost-benefit analysis	TBD	TBD
	Photovoice	TBD	TBD
	Baseline questionnaire		
	Endline questionnaire	TBD	TBD
	Endline qualitative Interviews	TBD	TBD

Phase 1: Diagnostics (Month 3- 14)

The aim of this phase is to map out and understand community knowledge, the systems, and services available at local level and everyday practices related to mental health. This is intended to identify the knowledge, practices, and resources available in the community and

For peer review only

the experiences and beliefs held by community actors about mental health, mental illness and practices of care. Data collection initiated in April 2021 and was completed April 2022 for stage one and two. Stage three remains ongoing. Specific aims, and procedures linked to this stage are as follows:

(1) *Assess local mental health systems capacities and capabilities in collaboration with service actors.* This stage involves three modes of data collection and engagement. First a review of existing mental health national interventions and their implementation and a Systematic Applied Policy Review of mental health national plans and policies currently in force. Second, involves motivated ethnographies [22] of local mental health services and community needs, with semi-structured interviews with service providers in each site. Third, includes focus groups with service providers, which are conducted online during the pandemic period. WhatsApp discussion groups are used as a platform to engage time-strapped institutional (psychologists, social workers) and community practitioners (including traditional healers) in both sites.[23] The implementation of these steps is currently on-going, having started in February 2021.

(2) *Explore community understandings of mental health, mental distress, and wellbeing strategies in one urban and one rural PDET territory.* This involves a qualitative investigation of local understandings drawing on focus groups discussions, word association tasks, a Tree of Life exercise which focuses on experiences and community resources linked to achieving good mental health and wellbeing. It will also draw from the motivated ethnography in each site. 12 focus groups discussions divided by gender and age are envisaged.

(3) *Work with local communities to evaluate appropriateness of standard mental health measures, using participatory methodologies.* Three standardised Mental health measures PHQ-9; WHO-5, and Warwick-Edinburgh wellbeing scale were selected as

For peer review only

potential screening tools to evaluate the impact of community designed activities. Initial team discussions with non-academic partners established the potential local appropriateness of the measures before they were discussed with community members. All measures have been standardised for use with Colombian or Spanish speaking participants [24–26]. Focus groups will provide an opportunity to complete group cognitive interviews to explore meaning and perceptions of measures [27]. This critical stage is informed by previous pilot work conducted in Colombia by members of our team [6,28]

(4) *Assess the cost of the standard mental health services basket offer of local health systems*. The scarcity of data in these areas will make this stage challenging, but we are envisaging the potential collection of data from three sources: motivated ethnography, document analysis and service provider interviews (n – 30). This will allow us to understand comparative costing for community led supports where possible.

Phase 2: Intervention: PLA cycles to improve mental health community services (Months 15-27)

The aim of this phase is to design and implement a community led group intervention to a) identify social drivers of mental health and priority conditions, b) create shared spaces for dialogue and understanding of mental health, mental distress, and wellbeing, identifying facilitators and barriers to collaborative processes of communication and action; and 3) establish priorities for action that improve community's access to mental health services in PDET territories.

Intervention design: The intervention design is grounded in a participatory theory of change process. Its first component is a participatory Theory of Change (TOC) workshop to involve large numbers of community members in the intervention co-design process.

For peer review only

Participants from each community with interest in the project and their children were invited to a daylong workshop in Florencia.

Drawing on preliminary analysis from the diagnostic phase, participatory activities are designed to facilitate real-time contributions to three main dimensions of the theory of change process: *identification of challenges, assumptions, and preconditions, short and long-term outcomes and impacts*, and *backward chaining*. Manual development was led by RAB and refined by the academic team members. The TOC workshop manual is available in supplementary materials, in English and Spanish. A summary of this process is provided in table 2.

For peer review only

Table 2.
Theory Of Change workshop structure

TOC session	Stage	Connection to TOC process	Activity to be conducted	Time allowance for activity	Number of facilitators required	Resources required
Session 1	Challenges that hinder good mental health and mental health services	Identify challenges, assumptions, and context	Building problem trees	2 hours	2-4	Tape recorder<br>Flip chart<br>Paper<br>Coloured marker pens<br>Flash cards with themes from FGDs (5 full sets)
Session 2	Ideal world that enables good mental health and mental health services	Identify long-term outputs, other outputs and pathways to change.	Storytelling of an ideal world	1.5 hours	2-4	Tape recorder<br>Flip chart<br>Paper<br>Coloured marker pens<br>Photocopy of exercise
Session 3	Identify interventions which could be used to improve mental health and mental health services	Identify intervention and additional contexts.	Mapping and intervention building	1 hour	2-4	Tape recorder<br>Cardboards<br>Paper<br>Coloured marker pens<br>Flashcards

The TOC workshop was run in December 2021 facilitated by senior project members community researchers. A total of 44 people attended, equally split between each study site.

14 of these participants also attended the FGDs in phase 1. The sessions were audio recorded and data was transcribed and analysed in Spanish. The academic members of the project team used this data alongside preliminary analyses of focus group data and the focused ethnography, to develop a working model of the theory of change. This was presented to the wider project team and community researchers, for evaluation and validation.

Based on the findings of the TOC process, we identified that a participatory, learning and action (PLA) approach to the intervention would be an ideal structure. PLA cycles have been used widely in other resource-limited settings but to the best of our knowledge, our study is the first to implement PLA cycles at scale for community mental health improvement in Colombia. For example, their use has contributed to improved health outcomes for diabetes in Bangladesh,[29] and maternal and child health in India,[30] and are currently being evaluated for improvement in under-5 pneumonia in Nigeria.[31] Crucially, our adaptation seeks to enhance links across groups that are historically opposed and limited by unequal access to power: community service providers, ex-combatants, internally displaced people, and host community members. The value of these types of linking interventions for health systems improvement are well documented elsewhere [32].

Based on community priorities identified in the TOC process, the proposed outcomes for the PLA intervention are as follows. We organise these into primary outcomes which we feel may be achieved in the short term, as well as longer term outcomes that could occur with longer running of PLA groups. :

Primary outcomes: (1) increased access to mental health acknowledge and information by community members; (2) improved feelings of belongingness and community cohesion and (3) improved perceptions of communication and relationships between practitioners and communities.

For peer review only

Long term outcomes: 1) improved recognition of the importance of good mental health to wider health and wellbeing, 2) reduction of stigma around mental illness and mental health, 3) young people's increased participation and communication in family life and community activities, 4) improved mental wellbeing, 5) improved experience of services (Respect, listening, communication).

Intervention structure: The PLA intervention itself is comprised of 4 stages, running across 13 sessions (Figure 1).

Stage one – knowledge building*,* is designed to provide community members with opportunities to develop new knowledge and understanding about mental health linked to the priority issues identified in the ToC workshop.

Stage two – from reflection to action, where participants will engage in a series of prioritisation and planning activities to identify a single challenge or focus and a plan for local action to address the issue. This stage will end with a community forum which creates a formal link between key actors in the local mental health infrastructure. Key actors were identified in the ToC and the ethnography and will be invited to engage in the community forums.

Stage three – implementation will focus on groups' implementation of their projects, and group led monitoring of the implementation process and the delivery of the planned activities. We will suggest the use of photography and video to help increase the accessibility of this process to community members.

Stage four – evaluation will include a formalised participatory evaluation of each PLA group's intervention, exploring any potential impact and efficacy in attaining the desired outcomes. Group members will be invited to participate in a photovoice project to achieve this. Phase four will also involve the election of community mental health champions. These individuals will become the focal points about mental health issues in their communities, combining with existing local infrastructure (such as health committees) in the long term. They

For peer review only

will complete additional training provided by the project (i.e WHO quality rights training, Community MH gap training), as well as training on facilitating future cycles of the group for those projects who which to continue. (See figure 1 below).

[Insert Figure 1. PLA Groups Intervention]

PLA group implementation

Group facilitator Training: Community researchers are also facilitators for PLA groups. They completed full day of training, delivered in 5 short modules. The first of which included basic information about the project and the use of the manual. The next 4 modules corresponded to each PLA phase outlining the objectives of each session and activities. To compensate for the short time period, the training programme was organised around role play activities, where facilitators completed all activities to be used within the intervention. Training also included a refresher on the processes for referrals (the same as used in phase one), and introduction to new data monitoring processes.

PLA groups development: Sessions will be delivered in a by-weekly schedule, aiming to approximate two 3-hour sessions per month, running for six months to complete one cycle. Delivery of sessions will be supported by regular supervision by a member of the research team, as well as bi-weekly meetings with all community researchers, where implementation issues will be discussed. Due to time constraints created by the pandemic and funding instability created by geopolitical contexts in the UK, the pilot study will be restricted to a single cycle.

Group intervention structure will be determined by relevance to local context. In La Montañita, given the close ties between community members, it is likely that men and women will work together in groups in some cases. In Florencia, groups will likely be divided by sex and in both contexts will be divided by age, with young people meeting separately.

For peer review only

Phase 3: Evaluation (Months 20-27)

At programme level, we will explore the acceptability, appropriateness, and feasibility of a PAR approach to establish platforms for community-led mental health systems strengthening. To evaluate this, we will hold monthly team meetings to discuss process and implementation challenges. We will also convene two workshops to discuss the strengths and weaknesses of the overall PAR approach and PLA intervention with team members and invited service delivery and community member representatives.

At the intervention level, we will explore standard process and outcome evaluation parameters as summarised in Table 3, in line with MRC Complex intervention guidelines. For our intervention, we will evaluate potential impact at the individual and community level, combining traditional academic evaluations of outcomes using standardised measures, exit qualitative interviews with 30 participants (15 per site), and community led evaluation methods – using photovoice methods.

For peer review only

Table 3.

Outcome evaluation parameters for PLA group intervention.

Item	Definition	Indicators	Target group	Frequency of collection	Person responsible	Source of data	Tool required	Data type
Acceptability	Satisfaction with the content and delivery of components	Experiences of sessions	PLA Participants	Once	Research Team	Endline interview	Topic guide	Qualitative
Appropriateness	Usefulness, relevance, suitability of component	Describing the intervention as useful	PLA Participants	Once	Research Team	Endline interview<br>Endline questionnaire	Topic guide and survey	Qualitative and quantitative
Feasibility	Suitability of component for routine implementation	Delivery of sessions	Community researchers	Once	Research Team	Endline interview with community researchers<br>Field diaries	Topic guide and field diaries	Mixed
Fidelity of delivery	Delivery of the component as intended	Number of sessions conducted	PLA Participants	Once	Community Researchers	Attendance registers	Attendance registers	Quantitative
		Content of sessions	Community researchers	Monthly	Community Researchers	Field diaries	Field diaries	Mixed
		Participatory-ness of the sessions		Monthly	Community Researchers	Field diaries	Field diaries	Qualitative
	Intervention reach	Number of attendees	PLA Participants	Weekly	Community Researchers	Attendance registers	Attendance registers	Quantitative
		Profile of participants	PLA Participants	Once	Community Researchers	Questionnaire	Questionnaire (demographic session)	Quantitative
Fidelity of receipt	User understandings and performance resulting from receipt of component	Community led intervention strategies	PLA Participants	Once	Research Team	Field diaries and endline interviews	Topic guide and field diaries	Mixed
		Photovoice activities	PLA Participants	Once	Community Researchers	Photovoice	FG discussions and images	Qualitative

For peer review only

For peer review only - http://bmjopen.bmj.com/site/about/guidelines.xhtml

At the individual level, we will measure impact using standardised measures tested and validated by the community in Phase 1. These measures are summarised in Table 4. Where standardised tools were not available, we developed specific items to explore dimensions of knowledge, behaviour and practices linked to mental health knowledge. This was informed by KAP studies in other areas[33] and a similar tool used by other large scale mental health studies.[34] To better understand community and systems-level impacts, we will also run simulations to assess the cost-benefit or the cost-effectiveness of the actions that are (a) implemented and (b) planned in Phase 2. When it makes sense to monetize and data is available, results will be monetized using current knowledge of different uses of time by young individuals (education, work, political engagement, working for their communities) in resource-constrained countries for the cost-benefit analysis. When not possible, cost-effectiveness analysis will be developed. Costs will be estimated using the baseline quantification of cost of health services in WP1, if possible. Together, these strategies evaluate the pathways, mechanisms, and resources required for promoting and improving mental health services and inform future questions to be considered in future trials and scaling up of our intervention.

Table 4.
PLA intervention - Endline evaluation measures

**Long term Outcomes**	**Indicator**	**Measure**
Improved experiences of mental health	Improved well being	WHO-5 (5 items)
reduced symptoms of mental ill health	Reduced symptoms of depression	PHQ-2 (2 items)
**Short term outcomes**	**Indicator**	**Measure**
Improved perceptions of quality of relationships between practitioners and communities	Increased willingness to seek treatment	Perceptions on different Service providers (5 items)
Improved feelings of belongingness and community cohesion	Increased sense of attachment to place/home	Sense of belonging and attachment to place [35] (14 items)

For peer review only

	Increased feelings of emotional and community support<br><br>Increased feelings of inclusion and acknowledgement in the community	World Bank Social Capital measure (17 items)
	Improved perception of individual and collective agency<br><br>Positive sense of self/identity	Possible selves questionnaire [36] (6 items)
Increased mental health literacy Knowledge Attitudes and Practices (KAP) questions	Increased mental health literacy	Depression symptom knowledge (5 items)
		Stress symptom knowledge (5 items)
		Substance misuse symptom knoweldge (5 items)
	Greater acceptance of others seeking treatment	3 items
	Helping others to seek treatment	2 items
	More positive perceptions of mental illness	1 item
Reduction of mental health stigma	More willingness to discuss/explore mental health needs in communities and families	RIBS reported behaviours subscale (4 items)

Sampling

Across the project two sampling strategies were used. For the diagnostic phase, purposive sampling ensured selection on the basis of participants' characteristics[37] in our case, in-depth knowledge of the context and local mental health services, from both potential service users' and providers' perspectives. Within this framework, we adopted a maximum variation approach, selecting across a broad spectrum of characteristics which included age, gender, and mental health status. This will support an in-depth understanding of the range of different groups who populate PDET communities ensuring saturation of contexts, through triangulation of data and experiences.[38]

For peer review only

Inclusion and exclusion criteria will be uniform across the programme. Inclusion criteria for community members will include a) place of residency (Florencia/La Montañita), reported by the participant as their home; b) age (16-25 years old and 26+ years); c) willingness to voluntarily participate (inform consent signed) and d) self-reported emotional distress experiences. Service provider sampling will include (a), working in a health provider setting or in a decision-making scenario related to the health field will be used in addition to the criteria used for community members as an inclusion criterion. Those with untreated mental health affections, people unable to give consent, people under 15 years old, and people unrelated to health providing systems and institution in the case of health representatives will not be eligible for participation in our study.

For the intervention, purposive sampling will be used to include community members who participated in the diagnostic phase as well as availability sampling to include a wide range of other community members. We did not conduct a formal sample size calculation due to the lack of data on the expected intervention effect size linked to our outcomes. However, simple power analyses linked to the use of scales such as the PHQ-9 indicate that a sample size of approximately 30 is required to show significance changes in pre-post testing. Notwithstanding, our recruitment aims were guided by previous experience of the research team applying this method in similar populations in Colombia [6] were the attrition rate was found to be around 42% among a similarly highly mobile and critical population. This is similar to other projects working with vulnerable and transient populations in PDET territories in Colombia (Idrobo et al, personal communication).

Data analysis

Qualitative data across all phases will be analysed using thematic network,[39] Reflexive,[40] or Framework analysis.[41] Thematic network analysis will be used to understand community perceptions of wellbeing and emotional distress, and local mental

For peer review only

health services. Other thematic analysis methods mentioned will be used for analysing data derived from the motivated ethnography, qualitative data from our evaluation, and in the policy review to identify primary topics regarding access and mental health services in Colombia, particularly in PDET municipalities. Collaborative data analysis strategies will be applied across all our project analysis, involving participants and community researchers in data analysis, verifying outputs and guaranteeing data validity.

Descriptive analysis and simple regression modelling will be performed on quantitative data from our evaluation questionnaire to evidence changes regarding mental health and wellbeing, and community level outcomes (social capital and social belonging) before and after our intervention. These changes will be captured comparing baseline and endline results following the completion of the intervention.

Data availability

Manuals in their finalised forms will be made available in English and Spanish on a project website. A fully anonymised pilot quantitative dataset will be uploaded through and open access data repository (ReShare) at the time of publication of our impact and results. Qualitative data will not be made publicly available given the small size of our study communities, the intimacy of people's experiences and narratives and the wider lack of trust among citizens about research processes.

Patient and Public Involvement

Because of the nature of PAR research and our overall co-production approach, this project is committed to public involvement. Community partner organisations were involved in the framing and development of the project from the outset (including funding application stages) and are involved in major planning and decision-making. Intervention design processes involve everyday citizens, or 'potential service users' during all phases. The theory of change

For peer review only

approach planned for this study is rooted in participant and public involvement, diverging from other approaches that involve a handful of patient representatives, or make us of previously collected data from wider communities. Instead, the stage will include people with previous experience of mental health services, family members, friends, and potential service users within the theory of change process.

ETHICS AND DISSEMINATION

Ethical approval has been obtained from two academic institutions. One in Colombia [2021-1393] and the UK [16127/005]. We will disseminate our work across academic, policy and community platforms. We will produce peer-reviewed publications and policy reports, alongside public communication activities such as workshops, short-films, infographics, and photography exhibitions to highlight community projects. A detailed communication strategy will be finalised based on collaborative agreement across our entire team and policy stakeholders.

AUTHORS CONTRIBUTIONS

Given the participatory nature of this project, authors contributed to many credit roles. They are outlined below:

Funding acquisition - RAB, MCD, SJ, DMC

Conceptualisation – All authors contributed equally to conceptualisation

Methodology – All authors contributed equally to methodology.

Data curation, investigation – LFD, NVSJ, MCM, MGG

Project administration: MGG, MCD, RAB, SJ, LF, NVSJ

For peer review only

Formal Analysis – MCD, MGG, SJ, LFD (phase one leads) RAB, LFD, MCD (phase two leads) RAB, DMC, DL (phase three leads). All other authors are supporting contributors across all phases

Supervision – RAB, SJ, MCD, DM

Writing original draft – RAB, MCD (equal leads of this manuscript)

Writing – review and editing – All authors

Funding Statement

This work is funded by an UKRI/ESRC Newton Award, grant number ES/V013211 and a MINCIENCIAS award, grant number 856-2020.

Competing interests' statement

Authors declare that they have no competing interests

REFERENCES

1 Semrau M, Alem A, Ayuso-Mateos JL, *et al.* Strengthening mental health systems in low- and middle-income countries: recommendations from the Emerald programme. *BJPsych Open* 2019;**5**:e73. doi:10.1192/bjo.2018.90

2 Burgess R. COVID-19 mental-health responses neglect social realities. *Nature* Published Online First: 4 May 2020. doi:10.1038/d41586-020-01313-9

3 Tamayo-Agudelo W, Bell V. Armed conflict and mental health in Colombia. *BJPsych Int* 2019;**16**:40–2. doi:10.1192/bji.2018.4

4 Cuartas Ricaurte J, Karim LL, Martínez Botero MA, *et al.* The invisible wounds of five decades of armed conflict: inequalities in mental health and their determinants in Colombia. *Int J Public Health* 2019;**64**:703–11. doi:10.1007/s00038-019-01248-7

For peer review only

5 Cepeda-Pérez A, Giraldo-Vargas AM, Gómez-Lizarazu DE, *et al.* Evaluación Programa de Atención Psicosocial y Salud Integral a Víctimas – PAPSIVI: Informe Final. Bogotá, D.C: 2020. https://www.minsalud.gov.co/sites/rid/Lists/BibliotecaDigital/RIDE/DE/PS/informe-final-evaluacion-resultados-papsivi-ps.pdf

6 Burgess RA, Fonseca L. Re-thinking recovery in post-conflict settings: Supporting the mental well-being of communities in Colombia. *Glob Public Health* 2020;**15**:200–19. doi:10.1080/17441692.2019.1663547

7 Montenegro CR, Cornish F. Historicising involvement: the visibility of user groups in the modernisation of the Chilean Mental Health System. *Crit Public Health* 2019;**29**:61–73. doi:10.1080/09581596.2017.1400659

8 Burgess RA, Jain S, Petersen I, *et al.* Social interventions: a new era for global mental health? *The Lancet Psychiatry* 2020;**7**:118–9. doi:10.1016/S2215-0366(19)30397-9

9 WHO. *Research for universal health coverage*. WHO 2013. https://www.who.int/publications/i/item/9789240690837

10 Kroc Institute for International Pease Studies. Tres años después de la firma del Acuerdo Final en Colombia: Hacia la transformación territorial. Diciembre 2018 a Noviembre 2019. 2020. http://peaceaccords.nd.edu/wp-content/uploads/2020/06/200630-Informe-4-resumen-final.pdf

11 Agencia de Renovación del Territorio. ABCÉ de los PDET: Programas de Desarrollo con Enfoque Territorial. 2021. https://portal.renovacionterritorio.gov.co/descargar.php?idFile=29067

12 Campbell C, Burgess R. The role of communities in advancing the goals of the Movement for Global Mental Health. *Transcult Psychiatry* 2012;**49**:379–95. doi:10.1177/1363461512454643

For peer review only

13 Nelson G, Prilleltensky I. *Community psychology : in pursuit of liberation and well-being*. Basingstoke: : Palgrave Macmillan 2010.

14 Lund C, Brooke-Sumner C, Baingana F, *et al.* Social determinants of mental disorders and the Sustainable Development Goals: a systematic review of reviews. *The Lancet Psychiatry* 2018;**5**:357–69. doi:10.1016/S2215-0366(18)30060-9

15 Rose-Clarke K, Gurung D, Brooke-Sumner C, *et al.* Rethinking research on the social determinants of global mental health. *The Lancet Psychiatry* 2020;**7**:659–62. doi:10.1016/S2215-0366(20)30134-6

16 Departamento Administrativo Nacional de Estadística (DANE). La información del DANE en la toma de decisiones regionales: Florencia - Caquetá. 2020. https://www.dane.gov.co/files/investigaciones/planes-departamentos-ciudades/201211-InfoDane-Florencia-Caqueta.pdf

17 Médicos sin Fronteras. Tres veces víctimas: Víctimas de la violencia, el silencio y el abandono Conflicto armado y salud mental en el departamento de Caquetá, Colombia. Florencia: 2010. https://www.acnur.org/fileadmin/Documentos/Publicaciones/2010/7372.pdf

18 Departamento Administrativo Nacional de Estadística (DANE). Principales indicadores del mercado laboral - Julio de 2022. Bogotá, D.C: 2022. https://www.dane.gov.co/files/investigaciones/boletines/ech/ech/bol_empleo_jul_22.pdf

19 Departamento Administrativo Nacional de Estadística (DANE). Fortalecimiento a la Atención Integral a Víctimas del Conflicto Armado en el Municipio de La Montañita. Florencia: 2020. https://lamontanitacaqueta.micolombiadigital.gov.co/sites/lamontanitacaqueta/content/files/000342/17089_2020184100008-victimas.pdf

For peer review only

20 Burgess RA, Choudary N. Time is on our side: operationalising 'phase zero' in coproduction of mental health services for marginalised and underserved populations in London. *Int J Public Adm* 2021;**44**:753–66. doi:10.1080/01900692.2021.1913748

21 Baum F, MacDougall C, Smith D. Participatory action research. *J Epidemiol Community Heal* 2006;**60**:854–7. doi:10.1136/jech.2004.028662

22 Burgess RA. Policy, power, stigma and silence: Exploring the complexities of a primary mental health care model in a rural South African setting. *Transcult Psychiatry* 2016;**53**:719–42. doi:10.1177/1363461516679056

23 Dedios Sanguineti MC, Martínes Gómez M, Guarín Á. Using WhatsApp to collect data on displaced Venezuelans, internally displaced populations, and host communities in Colombia during COVID-19 lockdowns. World Bank Blogs Dev. Peace. 2022.https://blogs.worldbank.org/dev4peace/using-whatsapp-collect-data-displaced-venezuelans-internally-displaced-populations-and (accessed 11 Oct 2022).

24 Cassiani-Miranda CA, Cuadros-Cruz AK, Torres-Pinzón H, *et al.* Validity of the Patient Health Questionnaire-9 (PHQ-9) for depression screening in adult primary care users in Bucaramanga, Colombia. *Rev Colomb Psiquiatr (English ed)* 2021;**50**:11–21. doi:10.1016/j.rcpeng.2019.09.002

25 Campo-Arias A, Miranda-Tapia GA, Cogollo Z, *et al.* Reproducibilidad del Índice de Bienestar General (WHO-5 WBI) en estudiantes adolescentes. *Salud Uninorte* 2015;**31**:18–24. https://www.redalyc.org/articulo.oa?id=81739659003

26 Serrani Azcurra D. Traducción, adaptación al español y validación de la escala de bienestar mental de WARWICK-EDINBURGH en una muestra de adultos mayores argentinos. *Acta Colomb Psicol* 2015;**18**:79–93. doi:10.14718/ACP.2015.18.1.8

27 Ouimet JA, Bunnage JC, Carini RM, *et al.* Using Focus Groups, Expert Advice, and Cognitive Interviews to Establish the Validity of a College Student Survey. *Res High*

For peer review only

Educ 2004;**45**:233–50.http://www.jstor.org/stable/40197291

28 Zamora-Moncayo E, Burgess RA, Fonseca L, *et al.* Gender, mental health and resilience in armed conflict: listening to life stories of internally displaced women in Colombia. *BMJ Glob Heal* 2021;**6**:e005770. doi:10.1136/bmjgh-2021-005770

29 Morrison J, Akter K, Jennings HM, *et al.* Participatory learning and action to address type 2 diabetes in rural Bangladesh: a qualitative process evaluation. *BMC Endocr Disord* 2019;**19**:118. doi:10.1186/s12902-019-0447-3

30 Seward N, Neuman M, Colbourn T, *et al.* Effects of women's groups practising participatory learning and action on preventive and care-seeking behaviours to reduce neonatal mortality: A meta-analysis of cluster-randomised trials. *PLOS Med* 2017;**14**:e1002467. doi:10.1371/journal.pmed.1002467

31 King C, Burgess RA, Bakare AA, *et al.* Integrated Sustainable childhood Pneumonia and Infectious disease Reduction in Nigeria (INSPIRING) through whole system strengthening in Jigawa, Nigeria: study protocol for a cluster randomised controlled trial. *Trials* 2022;**23**:95. doi:10.1186/s13063-021-05859-5

32 Durrance-Bagale A, Marzouk M, Tung LS, *et al.* Community engagement in health systems interventions and research in conflict-affected countries: a scoping review of approaches. *Glob Health Action* 2022;**15**. doi:10.1080/16549716.2022.2074131

33 Abrahams Z, Jacobs Y, Mohlamonyane M, *et al.* Implementation outcomes of a health systems strengthening intervention for perinatal women with common mental disorders and experiences of domestic violence in South Africa: Pilot feasibility and acceptability study. *BMC Health Serv Res* 2022;**22**:641. doi:10.1186/s12913-022-08050-x

34 Newson JJ, Thiagarajan TC. Assessment of Population Well-Being With the Mental Health Quotient (MHQ): Development and Usability Study. *JMIR Ment Heal*

For peer review only

2020;**7**:e17935. doi:10.2196/17935

35 Jovchelovitch S, Priego-Hernández J. *Underground Sociabilities: identity, culture and resistance in the favelas of Rio*. Brasilia, Paris: UNESCO 2013.

36 Oyserman D, Johnson E, James L. Seeing the Destination but Not the Path: Effects of Socioeconomic Disadvantage on School-focused Possible Self Content and Linked Behavioral Strategies. *Self Identity* 2011;**10**:474–92. doi:10.1080/15298868.2010.487651

37 Etikan I, Musa S, Akassim RS. Comparison of Convenience Sampling and Purposive Sampling. *Am J Theor Appl Stat* 2016;**5**:1. doi:10.11648/j.ajtas.20160501.11

38 Guest G, Bunce A, Johnson L. How Many Interviews Are Enough? *Field methods* 2006;**18**:59–82. doi:10.1177/1525822X05279903

39 Attride-stirling J. Thematic networks: an analytic tool for qualitative research. In: *Qualitative Research*. London; Thousand Oaks; New Delhi: : SAGE Publications 2001. 385–405.

40 Braun V, Clarke V. Reflecting on reflexive thematic analysis. *Qual Res Sport Exerc Heal* 2019;**11**:589–97. doi:10.1080/2159676X.2019.1628806

41 Gale NK, Heath G, Cameron E, *et al.* Using the framework method for the analysis of qualitative data in multi-disciplinary health research. *BMC Med Res Methodol* 2013;**13**:117. doi:10.1186/1471-2288-13-117

For peer review only

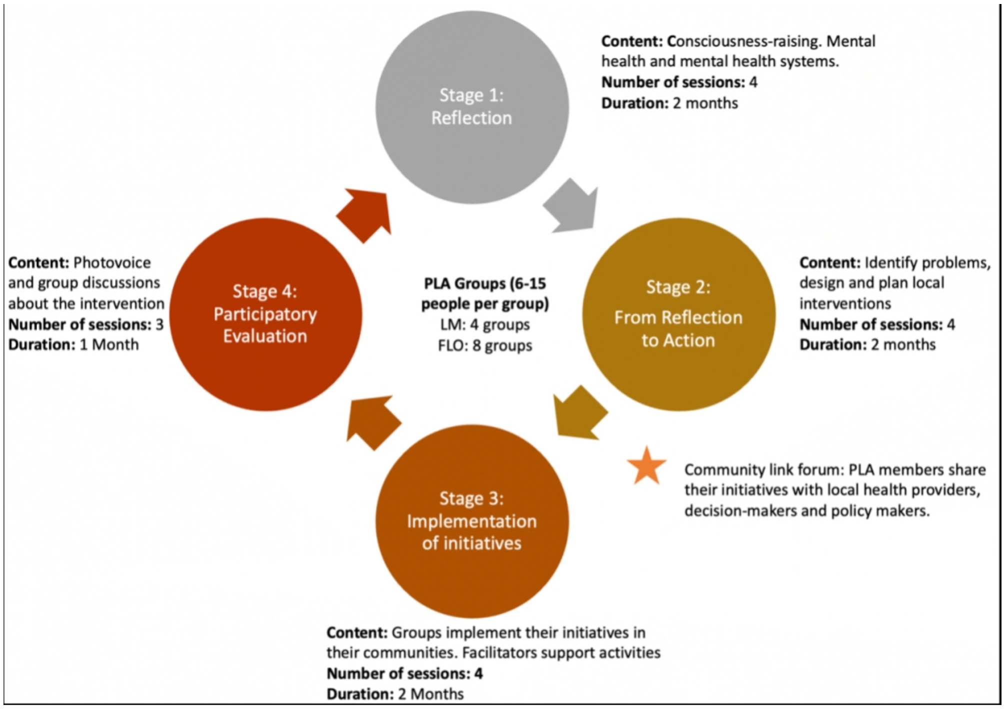

Figure 1 PLA intervention structure

159x112mm (220 x 220 DPI)

Starting From the Bottom: Building a Theory of Change (ToC) for community interventions to improve mental health services in PDET communities in Colombia

STARS-C project

Manual
English Version

For peer review only

Copyright © STARS-C project. Please contact project PI Dr. Rochelle Burgess prior to reproduction/reuse.
For peer review only - http://bmjopen.bmj.com/site/about/guidelines.xhtml

Theory of Change Workshop Manual

Methodology:

Conduct a public community forum and a Theory of Change workshop to collectively develop expectations, priorities and desired outcomes of mental health and mental health services for communities. This will also create an opportunity to set a broader goal for what people would like to see as the main outcomes of participation in through in this project.

Sampling:

50 participants

Procedure:

The below table provides a summary of what will be done in each session,and what the aim of each session is.

TOC session	Stage	Activity to be conducted	Time allowance for activity	# of facilitators required	Resources required
**Session 1**	Challenges that hinder good mental health and mental health services	Building problem trees	2 hours	2-4	1. Tape recorder<br>2. Flip chart<br>3. Paper<br>4. Coloured markerpens<br>5. Flash cards with themes from FGDs (5 full sets)
**Session 2**	Ideal world that enables good mental health and mental health services	Storytelling of an ideal world	3 hours	2-4	1. Tape recorder<br>2. Flip chart<br>3. Paper<br>4. Coloured markerpens<br>5. Photocopy of exercise
**Session 3**	Identify interventions which could be used to improve mental health and mental health services	Mapping and intervention building	1 hour	2-4	1. Tape recorder<br>2. Cardboards<br>3. Paper<br>4. Coloured markerpens<br>5. Flashcards

Copyright © STARS-C project. Please contact project PI Dr. Rochelle Burgess prior to reproduction/reuse.
For peer review only - http://bmjopen.bmj.com/site/about/guidelines.xhtml

Things to remember:

1. **Each session should be audio recorded to be transcribed/translated later.**
2. **You must make sure you take photos of all the outputs from each activity (e.g. problem tree etc).**

Introduction

We provided information about the project and the team, for participants to feel welcome and know who to ask if any questions should arise.
With the help of attendees, we developed a set of rules for respectful groups discussions and maintaining confidentiality.
Each participant was given a name tag, assigned a group number, and was sat on a table with the rest of their group. Facilitators prompted them to introduce themselves while activities started, as they would be working together throughout the day.

Session 1
Where we begin: Mapping and connecting factors that shape poor mental health

The aim of this session is to identify challenges that hinder good mental health and mental health services. We will do this, through using flash cards, which summarise the findings from our earlier focus group discussions, to build problem trees. When summarising the focus group discussions' data, be sure to avoid interpretations. The summary should be as much as possible a descriptive summary of raw data.

Step 1. *Brief introduction to the topic:* Remind participants of the activities during the FGDs and discuss the themes that emerged. You may want to facilitate a brief discussion to help warm up the room. For example, each facilitator is given a stack of randomized flash cards to distribute across the room. Then ask participants to place them into 'categories' on the walls.

Step 2. Divide participants into smaller groups. The groups should reflect the way that we will organize the PLA groups. Each group should have no more than 10 people.

Step 3: Assign the following topics to each group for them to create a problem tree.

1) Group of adults A (Florencia) – Mental health
2) Group of adults B (Florencia) – Mental health services
3) Group of young people (Florencia) – Mental Health
4) Group of adults A(La Montañita) – Mental health services
5) Group of adults B(La Montañita) – Mental health
6) Group of young people (La Montañita) – Mental health services

Step 4. Introduce the main activity – the problem tree (below) and provide instructions as follows:

Script: Today, we want to think deeply about the challenges that hinder good mental health and mental health services. We can articulate problems very clearly,

Copyright © STARS-C project. Please contact project PI Dr. Rochelle Burgess prior to reproduction/reuse.
For peer review only - http://bmjopen.bmj.com/site/about/guidelines.xhtml

but this task will help us to build connections between challenges at various levels in our lives. We can think of this more clearly, if we think about something physical in our environment, like a tree. A tree has different parts that all connect to make the whole. The roots, which are hidden, not always visible, but make it possible for the tree to exist. They grow first and have the largest effect. The main part of the tree – which is the trunk. It connects the roots to the outside world – it is the part that we see first, that is most visible. Finally, the leaves – the top of the tree, they grow up and out into the future.

The activity we will do first, is to build a problem tree, which helps us to make sense of these major themes that emerged from our focus group discussions. In your groups, you need to think about yourselves – as women, men, young people, and what specific problems matter the most to you, in your lives, and connect them from the 'roots' to the broader outcomes.

Each problem tree is split into three sections: the root (foundations/root causes) the core problem (what we can see) and the outcomes/consequences.

The roots are where you may map the root/hidden causes of challenges, such as unemployment, weak relationships; conflict; violence. The trunk signifies what the main problem is. For some people, this could be a mental health condition (depression), but it could be many other things as well (no education; isolation; hunger; family separation). Finally, at the branches, this signifies the outcomes, or the consequences of these difficulties. This could include things like: loss of work; low self-esteem; mental health challenges; exclusion, etc.

NB to facilitator: it may help, to build an example tree, while you are discussing these points above. You should have example flash cards to put in each part of the tree and ask participants where to put each.

Using the cards you have as a starting point, begin to build your problem trees. Some groups will make a tree for the experience of poor mental health, and the others will make a tree for what hinders mental health services. You will also be given blank cards, if there are things that were not captured in prior focus groups, but that you think are important to consider.

If it helps, you can imagine a person that you know, or that you have heard of, who is living through these issues right now. How would you build a tree to describe their life and experiences? How would you build a tree to describe their quest to seek treatment/support with the things they find difficult?

Instructions: Hand out cards to each group, showing the themes that emerged during the FGDs. Show participants the example problem tree below and give them 1 hour to discuss and create problem trees within their groups. In each group, provide a recorder device to capture the discussions being held by the participants.

Copyright © STARS-C project. Please contact project PI Dr. Rochelle Burgess prior to reproduction/reuse.
For peer review only - http://bmjopen.bmj.com/site/about/guidelines.xhtml

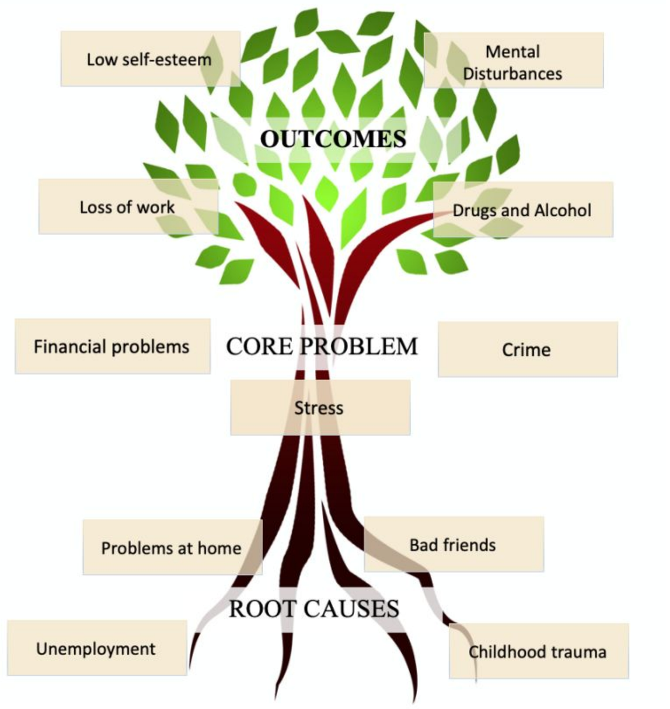

Step 5. After 1 hour, ask a representative from each group to share their problem tree with the rest of the participants (which should take approximately another hour).

Step 6. While the participants share their trees, one facilitator should be taking notes to support later analysis. Another should be taking more general notes to facilitate discussion. Note the similarities and differences between trees, and the challenges and outcomes of healthcare vs health services. These should be shared with the wider group, and participants should be asked for their thoughts on what is being shared.

Session 2
Storytelling of an ideal world – imagining outcomes and outputs

The aim of this session is to identify potential solutions to improve mental health services, and mental health outcomes. This is a long-term plan but should give participants a chance to think about what actions are required to achieve this long-term vision.

Step 1. Facilitators present the following phrase.

"The way we think about the future often focuses on the immediate future. However, when thinking is inspired by a vision, there is more room to achieve things which are thought of as 'unthinkable'. A vision for a better future gives us hope and increases motivation to take action to pursue that vision"

Copyright © STARS-C project. Please contact project PI Dr. Rochelle Burgess prior to reproduction/reuse.
For peer review only - http://bmjopen.bmj.com/site/about/guidelines.xhtml

Step 2. Participants should work in the same groups from activity 1.

Script: “Imagine your community 20 years from now. The national television agency (Día a Día/Séptimo Día) has prepared a programme on the outstanding achievements your community has made to increase the rates of access to mental health services and improving mental health in the community. The television/radio programme was prepared based on interviews with community members, local authorities, traditional leaders, and health institutions working in the district. Imagine what the programme would report about your community’s achievements in mental health. They have completed a special feature, on two people who have experienced this change. One person is someone whose mental health has been improved, and another is a practitioner who has worked with the patient and the community to build that change.

NB for facilitators: These questions should be handed out to each group on a piece of paper

General questions to consider for all parties

- ❑ What are major changes your community has made in the last 20 years to ensure good mental health in your community?
- ❑ What are the major changes your community has made in the last 20 years to increase the rates of access to mental health services?
- ❑ As a mental health provider, ¿what have you done to improve the mental health of your community?
    - Example: If you are a psychologist, how did you help your community?
- ❑ How have community leaders have supported efforts to address poor mental health?

Questions for your main characters:

- ❑ What actions did you do to start making life changes in terms of your mental health? Who was involved?
- ❑ What action plan did they follow in the first year to make the change happen?
- ❑ How did they convince other people who are important in their lives that this was the right decision?
- ❑ How did they keep going in the long run?

Scrip continued: “in your groups, you will need to write a story about this future world. It may help you to think about the questions in on the attached sheet of paper. You will present your story to the group in a role play (no more than 10 minutes long) of a television interview. There should be four speaking roles:

1) The journalist (who could be asking some of the questions we have provided)
2) a main character who has benefitted from the new world and services (could be the same person you thought about to help you do activity 1)
3) a health care provider
4) A key person who you feel is important to the story. (i.e could be a family member, a community leader, a politician, a friend, etc)

You will have 1 hour to work on this.

Copyright © STARS-C project. Please contact project PI Dr. Rochelle Burgess prior to reproduction/reuse.
For peer review only - http://bmjopen.bmj.com/site/about/guidelines.xhtml

Step **3**. After 1 hour, ask the groups to present their plays within each site. Then ask them to vote for the better story as this will be presented to the broader group including participants from the other site. After deciding which play to present, ask participants to add or improve their stories if they think they should.

Step 4. Finally, let participants present the play from each site to one another. The facilitators should be taking notes and asking people to think about similarities or make comments towards what is being presented after each play. Audio and video record each presentation and the plenary discussion for future analysis.

Session 3
Mapping and intervention building

The aim of this final session is to identify interventions which could be used to improve better mental health and mental health services in communities. It may be worth stating at this stage, that these discussions will shape how we run the second stage of our project – which are the activities we facilitate to improve mental health and improve relationships between mental health services and communities over the course of the next year.

Step 1. Divide participants into same groups as for previous activity

Step 2. Explain that they will need to think back to the problem trees from Activity 1 and what was discussed in Activity 2.

Step 3. Tell participants that they have 1 hour to consider these challenges and imagine possible solutions. Make cardboards with the following questions.

1. What are the interventions you need to improve mental health and mental health services in your community?
2. What resources do you need to implement those interventions/actions?
3. What are the expected outcomes of implementing those interventions?

Step 4: Ask participants to write down the answer to those questions in flashcards and then to paste them under each cardboard.

NB to facilitator: Register the answer provided. If there is enough time, share results with the broader group trying to highlight similarities and differences.

Closing statements

Participants were thanked for their time and contributions. Facilitators went around the room asking about people's experiences and any feedback for future activities.
Facilitators shared next-steps for the project to have a sense of continuity and stay in touch with the community.

Copyright © STARS-C project. Please contact project PI Dr. Rochelle Burgess prior to reproduction/reuse.
For peer review only - http://bmjopen.bmj.com/site/about/guidelines.xhtml

BMJ Open

Using Participatory Action Research to re-imagine community mental health services in Colombia: A Mixed-Methods Study Protocol

Journal:	*BMJ Open*
Manuscript ID	bmjopen-2022-069329.R1
Article Type:	Protocol
Date Submitted by the Author:	22-Nov-2022
Complete List of Authors:	Burgess, Rochelle; UCL Institute for Global Health<br>Dedios Sanguineti, María Cecilia; Universidad de Los Andes, School of Goernment<br>Maldonado-Carrizosa, Darío; Universidad de los Andes, School of Government<br>Fonseca, Laura; London School of Economics and Political Science, Department of Psychological and Behavioural Science<br>Vera San Juan, Norha; UCL Institute for Global Health; University College London, Rapid Research Appraisal and Evaluation Lab (RREAL)<br>Lucumí, Diego; Universidad de Los Andes, School of Government<br>González-Gort, Mónica; Universidad de Los Andes, School of Government<br>Melgar, Mónica Carreño; Universidad de los Andes, School of Government<br>Gaviria, María Fanny; Corporación Manigua<br>Tovar, Diego; Cooperativa multiactiva para el buen vivir y la paz del Caquetá<br>Jovchelovitch, Sandra; London School of Economics and Political Science, Department of Psychological and Behavioural Science
**Primary Subject Heading**:	Global health
Secondary Subject Heading:	Health services research, Mental health, Public health, Patient-centred medicine
Keywords:	MENTAL HEALTH, Depression & mood disorders < PSYCHIATRY, PUBLIC HEALTH

SCHOLARONE™
Manuscripts

Using Participatory Action Research to re-imagine community mental health services in Colombia: A Mixed-Methods Study Protocol

Corresponding author:

Rochelle A. Burgess,
Institute for Global Health,
30 Guilford Street
Rm. 3.04,
London
WC1N 1EH
r.burgess@ucl.ac.uk.

List of authors

Rochelle A. Burgess, Institute for Global Health, University College London, London, United Kingdom.
María Cecilia Dedios Sanguineti, School of Government, Universidad de Los Andes, Bogotá, Colombia.
Darío Maldonado-Carrizosa, School of Government, Universidad de Los Andes, Bogotá, Colombia.
Laura Fonseca, Department of Psychological and Behavioural Science, London School of Economics and Political Science, London, United Kingdom.
Norha Vera San Juan, Institute for Global Health, University College London, London, United Kingdom.
Diego Lucumí, School of Government, Universidad de Los Andes. Bogotá, Colombia.
Mónica González-Gort, School of Government, Universidad de Los Andes. Bogotá, Colombia.
Mónica Carreño Melgar, School of Government, Universidad de Los Andes, Bogotá, Colombia.
María Fanny Gaviria, Corporación Manigua (CORPOMANIGUA), Florencia, Colombia.
Diego Ferney Tovar, Cooperativa multiactiva para el buen vivir y la paz del Caquetá (COOMBUVIPAC), La Montañita, Colombia.
Sandra Jovchelovitch, Department of Psychological and Behavioural Science, London School of Economics and Political Science, London, United Kingdom.

Word count: 4,189

For peer review only

Key words

Colombia; mental health; health systems strengthening; community participation; community involvement

Authors and Institutions

Rochelle A. Burgess, r.burgess@ucl.ac.uk, University College London.
María Cecilia Dedios Sanguineti, m.dedios@uniandes.edu.co, Universidad de Los Andes.
Darío Maldonado-Carrizosa, dmaldonadoc@uniandes.edu.co, Universidad de Los Andes.
Laura Fonseca, l.m.fonseca-duran@lse.ac.uk, London School of Economics and Political Science.
Norha Vera San Juan, n.verasanjuan@ucl.ac.uk, University College London.
Diego Lucumí, di.lucumi@uniandes.edu.co, Universidad de Los Andes.
Mónica González-Gort, m.gonzalez22@uniandes.edu.co, Universidad de Los Andes.
Mónica Carreño Melgar, m.carrenom@uniandes.edu.co, Universidad de Los Andes.
María Fanny Gaviria, f.gaviria@corpomanigua.org, Corporación Manigua (CORPOMANIGUA).
Diego Ferney Tovar, pacercri@gmail.com, Cooperativa multiactiva para el buen vivir y la paz del Caquetá (COOMBUVIPAC).
Sandra Jovchelovitch, s.jovchelovitch@lse.ac.uk, London School of Economics and Political Science.

ORCID Ids

Rochelle A Burgess: 0000-0001-9749-7065
María Cecilia Dedios-Sanguineti: 0000-0002-6141-304X
Darío Maldonado-Carrizosa: 0000-0003-3544-208X
Laura Fonseca: 0000-0003-0638-3447
Norha Vera San Juan: 0000-0002-8677-7341
Diego Lucumí: 0000-0003-1834-7937
Mónica González-Gort: 0000-0002-5488-832X
Mónica Carreño Melgar: 0000-0003-2579-8568
María Fanny Gaviria: N/A (Non-academic partner)
Diego Ferney Tovar: N/A (Non-academic partner)
Sandra Jovchelovitch: 0000-0002-0073-2792

For peer review only

ABSTRACT

Introduction

Mental-health care systems are challenged by how they hear and respond to what marginalised communities experience as drivers of mental distress. In Colombia, this challenge intersects with wider challenges facing post-conflict reconstruction. Our pilot study will explore the feasibility and acceptability of a participatory approach to developing community-led participatory interventions for community mental health systems strengthening and mental health improvement, in two sites in Caquetá, Colombia.

Methods and analysis

The project is divided into three distinct phases aligned with community participatory action research (PAR) cycles: diagnostic, intervention, and evaluation. This allows us to use a participatory approach to design a community-led, bottom-up intervention for mental health systems strengthening and the promotion of mental health and wellbeing.

The diagnostic phase explores local understandings of mental health, mental distress, and access to mental health services from community members and health providers. The intervention stage will be guided by a participatory Theory of Change process. Community priorities will inform the development of a Participatory, Learning and action (PLA) informed group intervention, with a community linkage forum. The pilot of the PLA intervention will be evaluated using MRC process evaluation guidelines.

Ethics and dissemination

This project has received ethical approval from two sources. Universidad de Los Andes [2021-1393] and the University College London [16127/005]. Dissemination of findings will include academic publications, community forums, policy briefs, and visual media (cartoons, pod casts and short films).

For peer review only

Article summary - Strengths and limitations of this study

- This pilot study aims to provide evidence for a new methodology that meaningfully involves citizens developing and strengthening mental health systems in complex settings.
- The study pilots for the first time in Colombia participatory action research to design participatory learning and action groups (PLA) for improving mental health and strengthening community mental health systems.
- PLA groups will enable better collaboration between community knowledge systems, community members, and the services that are designed to support them, through 'community link' activities.
- The main challenge facing this pilot is the integration of participation across multiple sectors.
- PAR processes can be directly impacted by wider geopolitical realities – such as the UK government funding cuts, which disrupted community processes and relationship building.

For peer review only

INTRODUCTION

Globally, the burden of mental health conditions is shaped by gaps in services. In Low-Middle Income Countries (LMICs), 75% of the population lacks access to any form of care.[1] The COVID-19 pandemic exacerbated these challenges as intersecting social realities deepen distress, increase the incidence of mental health disorders, and overburden health systems.[2] In the case of Colombia, political violence, poverty, and displacement further aggravate this burden. Previous research shows that victims of armed conflict are more likely to suffer from mental health disorders,[3] with poverty explaining 86% of mental health inequalities in the country.[4]

Six years after the Peace Accords between the Colombian Government and the FARC guerrilla, there are still barriers in the implementation of the Psychosocial Care and Comprehensive Health Services for Victims programme (PAPSIVI) and the Psychosocial Wellbeing Component in the reintegration route for ex-combatants (Resolution n. 4309). In the case of ex-combatants, a dual status of victims and perpetrators requires balancing psychosocial well-being, personal protection, and political acceptability of mental health services. This population, like the victims of the conflict, reside in rural areas where services are scarce or non-existent.[3,5]

Mental-health care systems are challenged by how they hear and respond to what marginalised communities experience as drivers of mental distress.[6–8] This is acknowledged by global,[9] and national priorities, which call for providing accessible and quality services to overlooked communities. In Colombia, this includes territories and rural populations (campesinos) that are the focus of Territorially Focused Development Programmes (PDETs in Spanish), a national programme of development prioritising those who have been heavily affected by disproportionate armed conflict, poverty, illicit economies, and institutional fragility.[10,11]

For peer review only

Scaling-up services is important but only a partial response; sustainable solutions to improve mental health require dialogue between health systems and communities.[12] Community-owned and anchored interventions are critical to re-establishing trust between local populations and systems, particularly after periods of extended upheaval. In this context, integrating community-level experiences of mental health and mental distress with institutional responses by state-level actors is a necessary step towards effective community mental health services. This requires a multi-level interdisciplinary perspective that links individual and community well-being to wider institutional, socio-economic, and political contexts. Community Participatory Action Research (CPAR) approaches allow us to explore the ability to identify strengths and solutions produced by communities for communities, connecting them to wider systems, while acknowledging them as agents with the capacity to create effective, context sensitive solutions.[13]

As Colombia begins to refocus its efforts towards achieving these global and national policy aims, three critical areas require attention: 1) Wider social and political contextual factors that drive experiences of poor mental health,[14] 2) Increasing understanding of local embodied knowledge and lived experiences of communities and their relevance for building knowledge about mental health,[15] and 3) The role and resources offered by community participation in the co-design of interventions and services that are effective.[8]

In response to these demands, we will implement a participatory process to design, implement, and evaluate a participatory intervention to strengthen community mental health care systems in two PDET communities in Caquetá-Colombia. We are guided by the following research question: *what are the pathways, mechanisms, and resources needed to catalyse collaborative action between communities and institutions for promoting and improving mental health services for PDET communities?* To this end, we aim:

For peer review only

(1) To co-design and co-implement a participatory group intervention to create trust and opportunities for collaborative action between community and health system actors to improve the performance of community mental health services.

(2) To co-evaluate the group intervention in terms of process, outcomes (including individual and community mental health), and simulations of the cost-benefit and cost-effectiveness of the intervention at individual, community, and health services levels.

(3) To produce a manual based on the development, implementation, and evaluation of the intervention to guide communities and institutions in the application of these methods for developing and scaling up community mental health services in Colombia. We expect these tools to be made widely applicable in other low-resource or conflict-affected settings.

The project is divided into distinct phases aligned with community participatory action research (PAR) cycles reflecting diagnostic, intervention and evaluation. This protocol presents the STARS-C objectives, procedures, and methodological considerations for implementing a participatory mental health research project in conflict areas amidst the Covid-19 pandemic.

METHODS AND ANALYSIS

The project will be implemented in inter-related phases aligned with participatory action research (PAR). It will run from February 2021 to May 2023 in Caquetá, Colombia. Implementation of the group intervention will run from July 2022 – March 2023. The project has been co-designed through existing partnerships involving academics and two community-based organisations: (1) the Manigua Corporation [*Corpomanigua*], an organization of women with experience in the design and implementation of projects with marginalized communities, located in Florencia, representing an urban community and (2) the Multi-active Cooperative for Wellbeing and Peace of Caquetá (*Cooperativa Multiactiva para el Buen Vivir y la Paz del*

For peer review only

Caquetá- (COOMBUVIPAC), which represents a rural community of ex-combatants from the former guerrilla FARC-EP, located in the small village (*vereda*) Héctor Ramírez Poblado Center (CP-HR - former Territorial Space for Training and Reincorporation Héctor Ramírez) in the municipality of La Montañita.

Co-design and co-implementation will be further achieved through the appointment of community researchers (two from each site), who live and work in the communities being studied, and are not previously employed by our NGO partners. They will be involved in all stages of the implementation of the project as detailed below and were appointed prior to the drafting of this protocol. To ensure more equal partnerships in this work community researchers were trained in collecting qualitative information, quantitative questionnaires and in psychological first aid to support potential psychological and emotional distress among participants. Regular supervision is provided in real-time planned meetings. WhatsApp groups are utilised for constant communication.

Setting

Caquetá is one of Colombia's 32 departments, and the only region of the country in which all municipalities are included in the Territorially Focused Development Plans (PDET in Spanish). The project will be conducted in 2 of these PDET municipalities: Florencia and La Montañita. Each of the municipalities also represents diversity within a more general context of deprivation and adversity.

Florencia is Caquetá's capital city and constitutes its largest population with 173,011 inhabitants, [16]. Updated mental health statistics are not available at the municipality level; however, a report by MSF (2010) in Caquetá suggests that of the 60% of the nearly 5000 patients affected by armed conflict and internal displacement, 18% were diagnosed with adaptative disorders, 18% with relationship problems and problems associated with abuse or neglect , 11% by major depression with one episode, 9% with grief and 8% with mood

For peer review only

disorders [17]. Arguably, the prevalence of these mental health disorders relates to structural drivers such as high unemployment levels. According to the latest report done by the National Administrative Department of Statistics in 2020, the unemployment rate in Florencia was 25%, with women having a higher unemployment rate (29.2%) than men (21.5%),[16] both much more, than the current unemployment national rate of 11% [18]. As an urban area, Florencia has access to some specialized mental health facilities and staff, including psychologists, psychiatrists and nurses.

La Montañita is a rural area located to the south-west of Florencia and one of the areas most affected by the armed conflict, with 8,756 victims out of a total of 14,692 inhabitants[16,19]. No mental health statistics are available for the municipality but reports from local organisations point to mental distress associated with poverty and conflict as well unmet care needs. The project will be carried out in a small village (*vereda*) self-named *Centro Poblado Héctor Ramirez,* which is one of the former territorial spaces for training and reincorporation for former FARC-EP combatants (AETCR in Spanish) in La Montanita.

Design

The STARS-C programme outlines a three-phase process to guide stakeholders in the development and strengthening of community led mental health systems. It is informed by coproduction principles, to enable a platform for involving community members in a process of thinking through what changes are needed to improve access to, and the quality of mental health services[20]. Coproduction principles demand the inclusion of everyday actors, or potential service users, within processes of design and development. We will achieve this through involving everyday community members using a Community Participatory Action Research (CPAR) [21] model, to think through what changes are needed to improve access to and quality of mental health services[20]. As such the project combines participatory

For peer review only

qualitative inquiry across its three phases of diagnosis, intervention, and evaluation (see table 1) with quantitative assessments of mental health outcomes in a process described below. Our study builds on a pilot feasibility study of this approach in Cundinamarca-Colombia with a group of forty forcibly displaced persons.[6]

Table 1.
Phases and data collection strategies

Phase	Data Collection	Participants	
		La Montañita	Florencia
Diagnostic	Focus Group 1: Local understandings of mental health and mental distress- Tree of Life	n=42	n=57
	Focus Group 2: Evaluation of standardised measures of mental health	n=34	n=49
	Interviews Health Providers	n= 13	n=17
	Whatsapp focus groups Health Providers	n=11	n=10
	Motivated Ethnography (1 month)	Local Hospital-Community health post	City Hospital
Intervention Design	Theory of Change Workshop	N =25	n=25
Intervention implementation	PLA Groups-Stage 1: Reflection	4 groups	8 groups
	PLA Groups-Stage 2: From Reflection to action		
	PLA Groups-Stage 3: Implementation of initiatives		
	PLA Groups-Stage 4: Evaluation		
Evaluation	Cost-benefit analysis	TBD	TBD
	Photovoice		
	Baseline questionnaire		
	Endline questionnaire		
	Endline qualitative Interviews		

Phase 1: Diagnostics (Month 3- 14)

The aim of this phase is to map out and understand community knowledge, the systems, and services available at local level and everyday practices related to mental health. This is intended to identify the knowledge, practices, and resources available in the community and

For peer review only

the experiences and beliefs held by community actors about mental health, mental illness and practices of care. Data collection initiated in April 2021 and was completed April 2022 for stage one and two. Stage three remains ongoing. Specific aims, and procedures linked to this stage are as follows:

(1) *Assess local mental health systems capacities and capabilities in collaboration with service actors.* This stage involves three modes of data collection and engagement. First a review of existing mental health national interventions and their implementation and a Systematic Applied Policy Review of mental health national plans and policies currently in force. Second, involves motivated ethnographies [22] of local mental health services and community needs, with semi-structured interviews with service providers in each site. Third, includes focus groups with service providers, which are conducted online during the pandemic period. WhatsApp discussion groups are used as a platform to engage time-strapped institutional (psychologists, social workers) and community practitioners (including traditional healers) in both sites.[23] The implementation of these steps is currently on-going, having started in February 2021.

(2) *Explore community understandings of mental health, mental distress, and wellbeing strategies in one urban and one rural PDET territory.* This involves a qualitative investigation of local understandings drawing on focus groups discussions, word association tasks, a Tree of Life exercise which focuses on experiences and community resources linked to achieving good mental health and wellbeing. It will also draw from the motivated ethnography in each site. 12 focus groups discussions divided by gender and age are envisaged.

(3) *Work with local communities to evaluate appropriateness of standard mental health measures, using participatory methodologies.* Three standardised Mental health measures PHQ-9; WHO-5, and Warwick-Edinburgh wellbeing scale were selected as

For peer review only

potential screening tools to evaluate the impact of community designed activities. Initial team discussions with non-academic partners established the potential local appropriateness of the measures before they were discussed with community members. All measures have been standardised for use with Colombian or Spanish speaking participants [24–26]. Focus groups will provide an opportunity to complete group cognitive interviews to explore meaning and perceptions of measures [27]. This critical stage is informed by previous pilot work conducted in Colombia by members of our team [6,28]

(4) *Assess the cost of the standard mental health services basket offer of local health systems*. The scarcity of data in these areas will make this stage challenging, but we are envisaging the potential collection of data from three sources: motivated ethnography, document analysis and service provider interviews (n – 30). This will allow us to understand comparative costing for community led supports where possible.

Phase 2: Intervention: PLA cycles to improve mental health community services (Months 15-27)

The aim of this phase is to design and implement a community led group intervention to a) identify social drivers of mental health and priority conditions, b) create shared spaces for dialogue and understanding of mental health, mental distress, and wellbeing, identifying facilitators and barriers to collaborative processes of communication and action; and 3) establish priorities for action that improve community's access to mental health services in PDET territories.

Intervention design: The intervention design is grounded in a participatory theory of change process. Its first component is a participatory Theory of Change (TOC) workshop to involve large numbers of community members in the intervention co-design process.

For peer review only

Participants from each community with interest in the project and their children were invited to a daylong workshop in Florencia.

Drawing on preliminary analysis from the diagnostic phase, participatory activities are designed to facilitate real-time contributions to three main dimensions of the theory of change process: *identification of challenges, assumptions, and preconditions, short and long-term outcomes and impacts*, and *backward chaining*. Manual development was led by RAB and refined by the academic team members. The TOC workshop manual is available in supplementary materials, in English and Spanish. A summary of this process is provided in table 2.

Table 2.
Theory Of Change workshop structure

TOC session	Stage	Connection to TOC process	Activity to be conducted	Time allowance for activity	Number of facilitators required	Resources required
Session 1	Challenges that hinder good mental health and mental health services	Identify challenges, assumptions, and context	Building problem trees	2 hours	2-4	Tape recorder<br>Flip chart<br>Paper<br>Coloured marker pens<br>Flash cards with themes from FGDs (5 full sets)
Session 2	Ideal world that enables good mental health and mental health services	Identify long-term outputs, other outputs and pathways to change.	Storytelling of an ideal world	1.5 hours	2-4	Tape recorder<br>Flip chart<br>Paper<br>Coloured marker pens<br>Photocopy of exercise
Session 3	Identify interventions which could be used to improve mental health and mental health services	Identify intervention and additional contexts.	Mapping and intervention building	1 hour	2-4	Tape recorder<br>Cardboards<br>Paper<br>Coloured marker pens<br>Flashcards

The TOC workshop was run in December 2021 facilitated by senior project members community researchers. A total of 44 people attended, equally split between each study site.

For peer review only

14 of these participants also attended the FGDs in phase 1. The sessions were audio recorded and data was transcribed and analysed in Spanish. The academic members of the project team used this data alongside preliminary analyses of focus group data and the focused ethnography, to develop a working model of the theory of change. This was presented to the wider project team and community researchers, for evaluation and validation.

Based on the findings of the TOC process (see supplementary data for final TOC), we identified that a participatory, learning and action (PLA) approach to the intervention would be an ideal structure. PLA cycles have been used widely in other resource-limited settings but to the best of our knowledge, our study is the first to implement PLA cycles at scale for community mental health improvement in Colombia. For example, their use has contributed to improved health outcomes for diabetes in Bangladesh,[29] and maternal and child health in India,[30] and are currently being evaluated for improvement in under-5 pneumonia in Nigeria.[31] Crucially, our adaptation seeks to enhance links across groups that are historically opposed and limited by unequal access to power: community service providers, ex-combatants, internally displaced people, and host community members. The value of these types of linking interventions for health systems improvement are well documented elsewhere [32].

Based on community priorities identified in the TOC process, the proposed outcomes for the PLA intervention are as follows. We organise these into primary outcomes which we feel may be achieved in the short term, as well as longer term outcomes that could occur with longer running of PLA groups:

Primary outcomes: (1) increased access to mental health acknowledge and information by community members; (2) improved feelings of belongingness and community cohesion and (3) improved perceptions of communication and relationships between practitioners and communities.

For peer review only

Long term outcomes: 1) improved recognition of the importance of good mental health to wider health and wellbeing, 2) reduction of stigma around mental illness and mental health, 3) young people's increased participation and communication in family life and community activities, 4) improved mental wellbeing, 5) improved experience of services (Respect, listening, communication).

Intervention structure: The PLA intervention itself is comprised of 4 stages, running across 13 sessions (Figure 1).

***Stage one – knowledge building**,* is designed to provide community members with opportunities to develop new knowledge and understanding about mental health linked to the priority issues identified in the ToC workshop.

Stage two – from reflection to action, where participants will engage in a series of prioritisation and planning activities to identify a single challenge or focus and a plan for local action to address the issue. This stage will end with a community forum which creates a formal link between key actors in the local mental health infrastructure. Key actors were identified in the ToC and the ethnography and will be invited to engage in the community forums.

Stage three – implementation will focus on groups' implementation of their projects, and group led monitoring of the implementation process and the delivery of the planned activities. We will suggest the use of photography and video to help increase the accessibility of this process to community members.

Stage four – evaluation will include a formalised participatory evaluation of each PLA group's intervention, exploring any potential impact and efficacy in attaining the desired outcomes. Group members will be invited to participate in a photovoice project to achieve this. Phase four will also involve the election of community mental health champions. These individuals will become the focal points about mental health issues in their communities, combining with existing local infrastructure (such as health committees) in the long term. They

For peer review only

will complete additional training provided by the project (i.e WHO quality rights training, Community MH gap training), as well as training on facilitating future cycles of the group for those projects who which to continue. (See figure 1 below).

[Insert Figure 1. PLA Groups Intervention]

PLA group implementation

Group facilitator Training: Community researchers are also facilitators for PLA groups. They completed full day of training, delivered in 5 short modules. The first of which included basic information about the project and the use of the manual. The next 4 modules correspond to each PLA phase outlining the objectives of each session and activities. To compensate for the short time period, the training programme was organised around role play activities, where facilitators completed all activities to be used within the intervention. Training also included a refresher on the processes for referrals (the same as used in phase one), and introduction to new data monitoring processes.

PLA groups development: Sessions will be delivered in a by-weekly schedule, aiming to approximate two 3-hour sessions per month, running for six months to complete one cycle. Delivery of sessions will be supported by regular supervision by a member of the research team, as well as bi-weekly meetings with all community researchers, where implementation issues will be discussed. Due to time constraints created by the pandemic and funding instability created by geopolitical contexts in the UK, the pilot study will be restricted to a single cycle.

Group intervention structure will be determined by relevance to local context. In La Montañita, given the close ties between community members, it is likely that men and women will work together in groups in some cases. In Florencia, groups will likely be divided by sex and in both contexts will be divided by age, with young people meeting separately.

For peer review only

Phase 3: Evaluation (Months 20-27)

At programme level, we will explore the acceptability, appropriateness, and feasibility of a PAR approach to establish platforms for community-led mental health systems strengthening. To evaluate this, we will hold monthly team meetings to discuss process and implementation challenges. We will also convene two workshops to discuss the strengths and weaknesses of the overall PAR approach and PLA intervention with team members and invited service delivery and community member representatives.

At the intervention level, we will explore standard process and outcome evaluation parameters as summarised in Table 3, in line with MRC Complex intervention guidelines. For our intervention, we will evaluate potential impact at the individual and community level, combining traditional academic evaluations of outcomes using standardised measures, exit qualitative interviews with 30 participants (15 per site), and community led evaluation methods – using photovoice methods.

For peer review only

Table 3.

Outcome evaluation parameters for PLA group intervention.

Item	Definition	Indicators	Target group	Frequency of collection	Person responsible	Source of data	Tool required	Data type
Acceptability	Satisfaction with the content and delivery of components	Experiences of sessions	PLA Participants	Once	Research Team	Midline/Endline interview	Topic guide	Qualitative
Appropriateness	Usefulness, relevance, suitability of component	Describing the intervention as useful	PLA Participants	Once	Research Team	Endline interview<br>Endline questionnaire	Topic guide and survey	Qualitative and quantitative
Feasibility	Suitability of component for routine implementation	Delivery of sessions	Community researchers	Once	Research Team	Endline interview with community researchers<br>Field diaries	Topic guide and field diaries	Mixed
Fidelity of delivery	Delivery of the component as intended	Number of sessions conducted	PLA Participants	Once	Community Researchers	Attendance registers	Attendance registers	Quantitative
		Content of sessions	Community researchers	Monthly	Community Researchers	Field diaries	Field diaries	Mixed
		Participatory-ness of the sessions		Monthly	Community Researchers	Field diaries	Field diaries	Qualitative
	Intervention reach	Number of attendees	PLA Participants	Weekly	Community Researchers	Attendance registers	Attendance registers	Quantitative
		Profile of participants	PLA Participants	Once	Community Researchers	Questionnaire	Questionnaire (demographic session)	Quantitative
Fidelity of receipt	User understandings and performance resulting from receipt of component	Community led intervention strategies	PLA Participants	Once	Research Team	Field diaries and endline interviews	Topic guide and field diaries	Mixed
		Photovoice activities	PLA Participants	Once	Community Researchers	Photovoice	FG discussions and images	Qualitative

For peer review only

At the individual level, we will measure impact using standardised measures tested and validated by the community in Phase 1. These measures are summarised in Table 4. Where standardised tools were not available, we developed specific items to explore dimensions of knowledge, behaviour and practices linked to mental health. This was informed by KAP studies in other areas[33] and a similar tool used by other large scale mental health studies.[34] To better understand community and systems-level impacts, we will also run simulations to assess the cost-benefit or the cost-effectiveness of the actions that are (a) implemented and (b) planned in Phase 2. When it makes sense to monetize and data is available, results will be monetized using current knowledge of different uses of time by young individuals (education, work, political engagement, working for their communities) in resource-constrained countries for the cost-benefit analysis. When not possible, cost-effectiveness analysis will be developed. Costs will be estimated using the baseline quantification of cost of health services in WP1, if possible. Together, these strategies evaluate the pathways, mechanisms, and resources required for promoting and improving mental health services and inform future questions to be considered in future trials and scaling up of our intervention.

Table 4.
PLA intervention - outcome evaluation measures

**Long term Outcomes**	**Indicator**	**Measure**
Improved experiences of mental health	Improved well being	WHO-5 (5 items)
reduced symptoms of mental ill health	Reduced symptoms of depression	PHQ-2 (2 items)
**Short term outcomes**	**Indicator**	**Measure**
Improved perceptions of quality of relationships between practitioners and communities	Increased willingness to seek treatment	Perceptions on different Service providers (5 items)
Improved feelings of belongingness and community cohesion	Increased sense of attachment to place/home	Sense of belonging and attachment to place [35] (14 items)

For peer review only

	Increased feelings of emotional and community support<br><br>Increased feelings of inclusion and acknowledgement in the community	World Bank Social Capital measure (17 items)
	Improved perception of individual and collective agency<br><br>Positive sense of self/identity	Possible selves questionnaire [36] (6 items)
Increased mental health literacy Knowledge Attitudes and Practices (KAP) questions	Increased mental health literacy	Depression symptom knowledge (5 items)
		Stress symptom knowledge (5 items)
		Substance misuse symptom knowledge (5 items)
	Greater acceptance of others seeking treatment	3 items
	Helping others to seek treatment	2 items
	More positive perceptions of mental illness	1 item
Reduction of mental health stigma	More willingness to discuss/explore mental health needs in communities and families	RIBS reported behaviours subscale (4 items)

Sampling

Across the project two sampling strategies were used. For the diagnostic phase, purposive sampling ensured selection on the basis of participants' characteristics[37] in our case, in-depth knowledge of the context and local mental health services, from both potential service users' and providers' perspectives. Within this framework, we adopted a maximum variation approach, selecting across a broad spectrum of characteristics which included age, gender, and mental health status. This will support an in-depth understanding of the range of different groups who populate PDET communities ensuring saturation of contexts, through triangulation of data and experiences.[38]

For peer review only

Inclusion and exclusion criteria will be uniform across the programme. Inclusion criteria for community members will include a) place of residency (Florencia/La Montañita), reported by the participant as their home; b) age (16-25 years old and 26+ years); c) willingness to voluntarily participate (inform consent signed) and d) self-reported emotional distress experiences. Service provider sampling will include (a), working in a health provider setting or in a decision-making scenario related to the health field will be used in addition to the criteria used for community members as an inclusion criterion. Those with untreated mental health affections, people unable to give consent, people under 16 years old, and people unrelated to health providing systems and institution in the case of health representatives will not be eligible for participation in our study.

For the intervention, purposive sampling will be used to include community members who participated in the diagnostic phase as well as availability sampling to include a wide range of other community members. We did not conduct a formal sample size calculation due to the lack of data on the expected intervention effect size linked to our outcomes. However, simple power analyses linked to the use of scales such as the PHQ-9 indicate that a sample size of approximately 30 is required to show significance changes in pre-post testing. Notwithstanding, our recruitment aims were guided by previous experience of the research team applying this method in similar populations in Colombia [6] where the attrition rate was found to be around 42% among a similarly highly mobile and critical population. This is similar to other projects working with vulnerable and transient populations in PDET territories in Colombia (Idrobo et al, personal communication).

Data analysis

Qualitative data across all phases will be analysed using thematic network,[39] Reflexive,[40] or Framework analysis.[41] Thematic network analysis will be used to understand community perceptions of wellbeing and emotional distress, and local mental

For peer review only

health services. Other thematic analysis methods mentioned will be used for analysing data derived from the motivated ethnography, qualitative data from our evaluation, and in the policy review to identify primary topics regarding access and mental health services in Colombia, particularly in PDET municipalities. Collaborative data analysis strategies will be applied across all our project analysis, involving participants and community researchers in data analysis, verifying outputs and guaranteeing data validity.

Descriptive analysis and simple regression modelling will be performed on quantitative data from our evaluation questionnaire to evidence changes regarding mental health and wellbeing, and community level outcomes (social capital and social belonging) before and after our intervention. These changes will be captured comparing baseline and endline results following the completion of the intervention.

Data availability

Manuals in their finalised forms will be made available in English and Spanish on a project website. A fully anonymised pilot quantitative dataset will be uploaded through and open access data repository (ReShare) at the time of publication of our impact and results. Qualitative data will not be made publicly available given the small size of our study communities, the intimacy of people's experiences and narratives, and the wider lack of trust among citizens about research processes.

Patient and Public Involvement

Because of the nature of PAR research and our overall co-production approach, this project is committed to public involvement. Community partner organisations were involved in the framing and development of the project from the outset (including funding application stages) and are involved in major planning and decision-making. Intervention design processes involve everyday citizens, or 'potential service users' during all phases. The theory of change

For peer review only

approach planned for this study is rooted in participant and public involvement, diverging from other approaches that involve a handful of patient representatives, or make use of previously collected data from wider communities. Instead, the stage will include people with previous experience of mental health services, family members, friends, and potential service users within the theory of change process.

ETHICS AND DISSEMINATION

Ethical approval has been obtained from two academic institutions. One in Colombia [2021-1393] and the UK [16127/005]. We will disseminate our work across academic, policy and community platforms. We will produce peer-reviewed publications and policy reports, alongside public communication activities such as workshops, short-films, infographics, and photography exhibitions to highlight community projects. A detailed communication strategy will be finalised based on collaborative agreement across our entire team and policy stakeholders.

AUTHORS CONTRIBUTIONS

Given the participatory nature of this project, authors contributed to many credit roles. They are outlined as follows. Writing original draft: RAB and MCDS are equal first authors on this manuscript. All other authors contributed to reviewing and editing of this manuscript. All authors contributed to the conceptualisation and methodology of the project. Funding acquisition was led by RAB, MCDS, SJ, DMC, MFG and DFT. Project administration is led by MGG, MCDS, RAB, SJ, LF, NVSJ. Data curation, investigation is led by LF, NVSJ, MCM, MGG. Formal Analysis: MCDS, MGG, SJ, LF, are phase one analysis leads. RAB, LF, MCDS are phase two leads. RAB, DMC, DL are phase three leads. All other authors

For peer review only

supporting analysis contributors across all phases. Supervision across this project is completed by RAB, SJ, MCDS, DMC.

Funding Statement

This work is funded by an UKRI/ESRC Newton Award, grant number ES/V013211 and a MINCIENCIAS award, grant number 856-2020.

Competing interests' statement

Authors declare that they have no competing interests

For peer review only

REFERENCES

1 Semrau M, Alem A, Ayuso-Mateos JL, *et al.* Strengthening mental health systems in low- and middle-income countries: recommendations from the Emerald programme. *BJPsych Open* 2019;**5**:e73. doi:10.1192/bjo.2018.90

2 Burgess R. COVID-19 mental-health responses neglect social realities. *Nature* Published Online First: 4 May 2020. doi:10.1038/d41586-020-01313-9

3 Tamayo-Agudelo W, Bell V. Armed conflict and mental health in Colombia. *BJPsych Int* 2019;**16**:40–2. doi:10.1192/bji.2018.4

4 Cuartas Ricaurte J, Karim LL, Martínez Botero MA, *et al.* The invisible wounds of five decades of armed conflict: inequalities in mental health and their determinants in Colombia. *Int J Public Health* 2019;**64**:703–11. doi:10.1007/s00038-019-01248-7

5 Cepeda-Pérez A, Giraldo-Vargas AM, Gómez-Lizarazu DE, *et al.* Evaluación Programa de Atención Psicosocial y Salud Integral a Víctimas – PAPSIVI: Informe Final. Bogotá, D.C: 2020. https://www.minsalud.gov.co/sites/rid/Lists/BibliotecaDigital/RIDE/DE/PS/informe-

final-evaluacion-resultados-papsivi-ps.pdf

6 Burgess RA, Fonseca L. Re-thinking recovery in post-conflict settings: Supporting the mental well-being of communities in Colombia. *Glob Public Health* 2020;**15**:200–19. doi:10.1080/17441692.2019.1663547

7 Montenegro CR, Cornish F. Historicising involvement: the visibility of user groups in the modernisation of the Chilean Mental Health System. *Crit Public Health* 2019;**29**:61–73. doi:10.1080/09581596.2017.1400659

8 Burgess RA, Jain S, Petersen I, *et al.* Social interventions: a new era for global mental health? *The Lancet Psychiatry* 2020;**7**:118–9. doi:10.1016/S2215-0366(19)30397-9

9 WHO. *Research for universal health coverage*. WHO 2013. https://www.who.int/publications/i/item/9789240690837

10 Kroc Institute for International Pease Studies. Tres años después de la firma del Acuerdo Final en Colombia: Hacia la transformación territorial. Diciembre 2018 a Noviembre 2019. 2020. http://peaceaccords.nd.edu/wp-content/uploads/2020/06/200630-Informe-4-resumen-final.pdf

11 Agencia de Renovación del Territorio. ABCÉ de los PDET: Programas de Desarrollo con Enfoque Territorial. 2021. https://portal.renovacionterritorio.gov.co/descargar.php?idFile=29067

12 Campbell C, Burgess R. The role of communities in advancing the goals of the Movement for Global Mental Health. *Transcult Psychiatry* 2012;**49**:379–95. doi:10.1177/1363461512454643

13 Nelson G, Prilleltensky I. *Community psychology : in pursuit of liberation and well-being*. Basingstoke: : Palgrave Macmillan 2010.

14 Lund C, Brooke-Sumner C, Baingana F, *et al.* Social determinants of mental disorders and the Sustainable Development Goals: a systematic review of reviews. *The Lancet*

For peer review only

Psychiatry 2018;**5**:357–69. doi:10.1016/S2215-0366(18)30060-9

15 Rose-Clarke K, Gurung D, Brooke-Sumner C, *et al.* Rethinking research on the social determinants of global mental health. *The Lancet Psychiatry* 2020;**7**:659–62. doi:10.1016/S2215-0366(20)30134-6

16 Departamento Administrativo Nacional de Estadística (DANE). La información del DANE en la toma de decisiones regionales: Florencia - Caquetá. 2020. https://www.dane.gov.co/files/investigaciones/planes-departamentos-ciudades/201211-InfoDane-Florencia-Caqueta.pdf

17 Médicos sin Fronteras. Tres veces víctimas: Víctimas de la violencia, el silencio y el abandono Conflicto armado y salud mental en el departamento de Caquetá, Colombia. Florencia: 2010. https://www.acnur.org/fileadmin/Documentos/Publicaciones/2010/7372.pdf

18 Departamento Administrativo Nacional de Estadística (DANE). Principales indicadores del mercado laboral - Julio de 2022. Bogotá, D.C: 2022. https://www.dane.gov.co/files/investigaciones/boletines/ech/ech/bol_empleo_jul_22.pdf

19 Departamento Administrativo Nacional de Estadística (DANE). Fortalecimiento a la Atención Integral a Víctimas del Conflicto Armado en el Municipio de La Montañita. Florencia: 2020. https://lamontanitacaqueta.micolombiadigital.gov.co/sites/lamontanitacaqueta/content/files/000342/17089_2020184100008-victimas.pdf

20 Burgess RA, Choudary N. Time is on our side: operationalising 'phase zero' in coproduction of mental health services for marginalised and underserved populations in London. *Int J Public Adm* 2021;**44**:753–66. doi:10.1080/01900692.2021.1913748

21 Baum F, MacDougall C, Smith D. Participatory action research. *J Epidemiol*

For peer review only

Community Heal 2006;**60**:854–7. doi:10.1136/jech.2004.028662

22 Burgess RA. Policy, power, stigma and silence: Exploring the complexities of a primary mental health care model in a rural South African setting. *Transcult Psychiatry* 2016;**53**:719–42. doi:10.1177/1363461516679056

23 Dedios Sanguineti MC, Martínes Gómez M, Guarín Á. Using WhatsApp to collect data on displaced Venezuelans, internally displaced populations, and host communities in Colombia during COVID-19 lockdowns. World Bank Blogs Dev. Peace. 2022.https://blogs.worldbank.org/dev4peace/using-whatsapp-collect-data-displaced-venezuelans-internally-displaced-populations-and (accessed 11 Oct 2022).

24 Cassiani-Miranda CA, Cuadros-Cruz AK, Torres-Pinzón H, *et al.* Validity of the Patient Health Questionnaire-9 (PHQ-9) for depression screening in adult primary care users in Bucaramanga, Colombia. *Rev Colomb Psiquiatr (English ed)* 2021;**50**:11–21. doi:10.1016/j.rcpeng.2019.09.002

25 Campo-Arias A, Miranda-Tapia GA, Cogollo Z, *et al.* Reproducibilidad del Índice de Bienestar General (WHO-5 WBI) en estudiantes adolescentes. *Salud Uninorte* 2015;**31**:18–24. https://www.redalyc.org/articulo.oa?id=81739659003

26 Serrani Azcurra D. Traducción, adaptación al español y validación de la escala de bienestar mental de WARWICK-EDINBURGH en una muestra de adultos mayores argentinos. *Acta Colomb Psicol* 2015;**18**:79–93. doi:10.14718/ACP.2015.18.1.8

27 Ouimet JA, Bunnage JC, Carini RM, *et al.* Using Focus Groups, Expert Advice, and Cognitive Interviews to Establish the Validity of a College Student Survey. *Res High Educ* 2004;**45**:233–50.http://www.jstor.org/stable/40197291

28 Zamora-Moncayo E, Burgess RA, Fonseca L, *et al.* Gender, mental health and resilience in armed conflict: listening to life stories of internally displaced women in Colombia. *BMJ Glob Heal* 2021;**6**:e005770. doi:10.1136/bmjgh-2021-005770

For peer review only

29 Morrison J, Akter K, Jennings HM, *et al.* Participatory learning and action to address type 2 diabetes in rural Bangladesh: a qualitative process evaluation. *BMC Endocr Disord* 2019;**19**:118. doi:10.1186/s12902-019-0447-3

30 Seward N, Neuman M, Colbourn T, *et al.* Effects of women's groups practising participatory learning and action on preventive and care-seeking behaviours to reduce neonatal mortality: A meta-analysis of cluster-randomised trials. *PLOS Med* 2017;**14**:e1002467. doi:10.1371/journal.pmed.1002467

31 King C, Burgess RA, Bakare AA, *et al.* Integrated Sustainable childhood Pneumonia and Infectious disease Reduction in Nigeria (INSPIRING) through whole system strengthening in Jigawa, Nigeria: study protocol for a cluster randomised controlled trial. *Trials* 2022;**23**:95. doi:10.1186/s13063-021-05859-5

32 Durrance-Bagale A, Marzouk M, Tung LS, *et al.* Community engagement in health systems interventions and research in conflict-affected countries: a scoping review of approaches. *Glob Health Action* 2022;**15**. doi:10.1080/16549716.2022.2074131

33 Abrahams Z, Jacobs Y, Mohlamonyane M, *et al.* Implementation outcomes of a health systems strengthening intervention for perinatal women with common mental disorders and experiences of domestic violence in South Africa: Pilot feasibility and acceptability study. *BMC Health Serv Res* 2022;**22**:641. doi:10.1186/s12913-022-08050-x

34 Newson JJ, Thiagarajan TC. Assessment of Population Well-Being With the Mental Health Quotient (MHQ): Development and Usability Study. *JMIR Ment Heal* 2020;**7**:e17935. doi:10.2196/17935

35 Jovchelovitch S, Priego-Hernández J. *Underground Sociabilities: identity, culture and resistance in the favelas of Rio*. Brasilia, Paris: UNESCO 2013.

36 Oyserman D, Johnson E, James L. Seeing the Destination but Not the Path: Effects of

For peer review only

Socioeconomic Disadvantage on School-focused Possible Self Content and Linked Behavioral Strategies. *Self Identity* 2011;**10**:474–92. doi:10.1080/15298868.2010.487651

37 Etikan I, Musa S, Akassim RS. Comparison of Convenience Sampling and Purposive Sampling. *Am J Theor Appl Stat* 2016;**5**:1. doi:10.11648/j.ajtas.20160501.11

38 Guest G, Bunce A, Johnson L. How Many Interviews Are Enough? *Field methods* 2006;**18**:59–82. doi:10.1177/1525822X05279903

39 Attride-stirling J. Thematic networks: an analytic tool for qualitative research. In: *Qualitative Research*. London; Thousand Oaks; New Delhi: : SAGE Publications 2001. 385–405.

40 Braun V, Clarke V. Reflecting on reflexive thematic analysis. *Qual Res Sport Exerc Heal* 2019;**11**:589–97. doi:10.1080/2159676X.2019.1628806

41 Gale NK, Heath G, Cameron E, *et al.* Using the framework method for the analysis of qualitative data in multi-disciplinary health research. *BMC Med Res Methodol* 2013;**13**:117. doi:10.1186/1471-2288-13-117

For peer review only

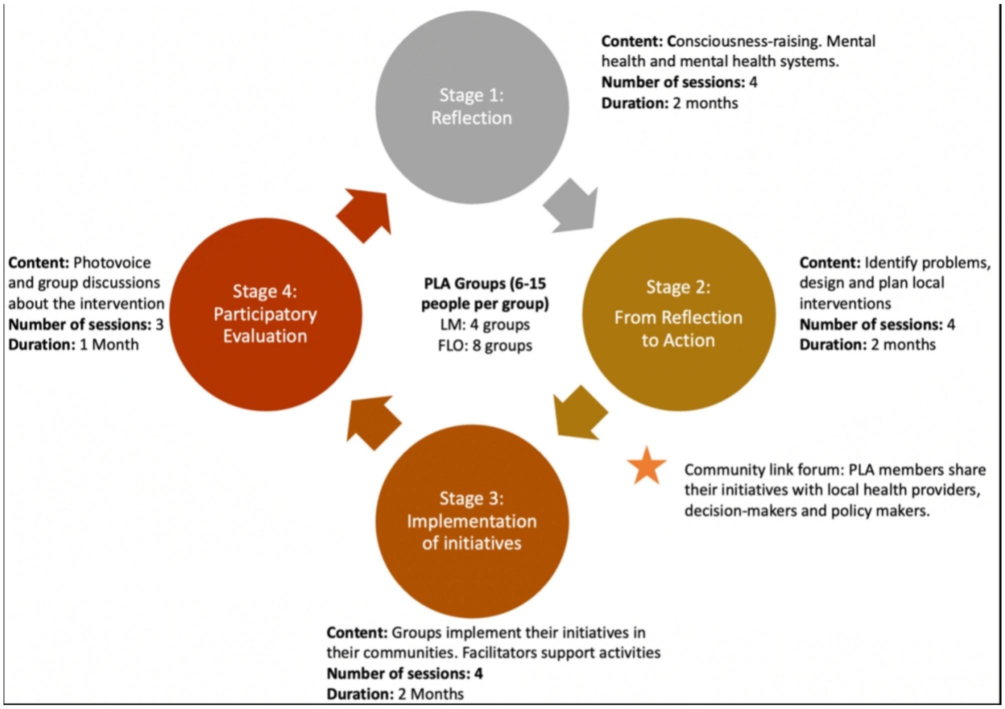

Figure 1 PLA intervention structure

159x112mm (220 x 220 DPI)

Starting From the Bottom: Building a Theory of Change (ToC) for community interventions to improve mental health services in PDET communities in Colombia

STARS-C project

Manual
English Version

For peer review only

Copyright © STARS-C project. Please contact project PI Dr. Rochelle Burgess prior to reproduction/reuse.
For peer review only - http://bmjopen.bmj.com/site/about/guidelines.xhtml

Theory of Change Workshop Manual

Methodology:

Conduct a public community forum and a Theory of Change workshop to collectively develop expectations, priorities and desired outcomes of mental health and mental health services for communities. This will also create an opportunity to set a broader goal for what people would like to see as the main outcomes of participation in through in this project.

Sampling:

50 participants

Procedure:

The below table provides a summary of what will be done in each session, and what the aim of each session is.

TOC session	Stage	Activity to be conducted	Time allowance for activity	# of facilitators required	Resources required
**Session 1**	Challenges that hinder good mental health and mental health services	Building problem trees	2 hours	2-4	1. Tape recorder<br>2. Flip chart<br>3. Paper<br>4. Coloured markerpens<br>5. Flash cards with themes from FGDs (5 full sets)
**Session 2**	Ideal world that enables good mental health and mental health services	Storytelling of an ideal world	3 hours	2-4	1. Tape recorder<br>2. Flip chart<br>3. Paper<br>4. Coloured markerpens<br>5. Photocopy of exercise
**Session 3**	Identify interventions which could be used to improve mental health and mental health services	Mapping and intervention building	1 hour	2-4	1. Tape recorder<br>2. Cardboards<br>3. Paper<br>4. Coloured markerpens<br>5. Flashcards

Copyright © STARS-C project. Please contact project PI Dr. Rochelle Burgess prior to reproduction/reuse.

For peer review only - http://bmjopen.bmj.com/site/about/guidelines.xhtml

Things to remember:

1. **Each session should be audio recorded to be transcribed/translated later.**
2. **You must make sure you take photos of all the outputs from each activity (e.g. problem tree etc).**

Introduction

We provided information about the project and the team, for participants to feel welcome and know who to ask if any questions should arise.
With the help of attendees, we developed a set of rules for respectful groups discussions and maintaining confidentiality.
Each participant was given a name tag, assigned a group number, and was sat on a table with the rest of their group. Facilitators prompted them to introduce themselves while activities started, as they would be working together throughout the day.

Session 1
Where we begin: Mapping and connecting factors that shape poor mental health

The aim of this session is to identify challenges that hinder good mental health and mental health services. We will do this, through using flash cards, which summarise the findings from our earlier focus group discussions, to build problem trees. When summarising the focus group discussions' data, be sure to avoid interpretations. The summary should be as much as possible a descriptive summary of raw data.

Step 1. *Brief introduction to the topic:* Remind participants of the activities during the FGDs and discuss the themes that emerged. You may want to facilitate a brief discussion to help warm up the room. For example, each facilitator is given a stack of randomized flash cards to distribute across the room. Then ask participants to place them into 'categories' on the walls.

Step 2. Divide participants into smaller groups. The groups should reflect the way that we will organize the PLA groups. Each group should have no more than 10 people.

Step 3: Assign the following topics to each group for them to create a problem tree.

1) Group of adults A (Florencia) – Mental health
2) Group of adults B (Florencia) – Mental health services
3) Group of young people (Florencia) – Mental Health
4) Group of adults A(La Montañita) – Mental health services
5) Group of adults B(La Montañita) – Mental health
6) Group of young people (La Montañita) – Mental health services

Step 4. Introduce the main activity – the problem tree (below) and provide instructions as follows:

Script: Today, we want to think deeply about the challenges that hinder good mental health and mental health services. We can articulate problems very clearly,

Copyright © STARS-C project. Please contact project PI Dr. Rochelle Burgess prior to reproduction/reuse.

but this task will help us to build connections between challenges at various levels in our lives. We can think of this more clearly, if we think about something physical in our environment, like a tree. A tree has different parts that all connect to make the whole. The roots, which are hidden, not always visible, but make it possible for the tree to exist. They grow first and have the largest effect. The main part of the tree – which is the trunk. It connects the roots to the outside world – it is the part that we see first, that is most visible. Finally, the leaves – the top of the tree, they grow up and out into the future.

The activity we will do first, is to build a problem tree, which helps us to make sense of these major themes that emerged from our focus group discussions. In your groups, you need to think about yourselves – as women, men, young people, and what specific problems matter the most to you, in your lives, and connect them from the 'roots' to the broader outcomes.

Each problem tree is split into three sections: the root (foundations/root causes) the core problem (what we can see) and the outcomes/consequences.

The roots are where you may map the root/hidden causes of challenges, such as unemployment, weak relationships; conflict; violence. The trunk signifies what the main problem is. For some people, this could be a mental health condition (depression), but it could be many other things as well (no education; isolation; hunger; family separation). Finally, at the branches, this signifies the outcomes, or the consequences of these difficulties. This could include things like: loss of work; low self-esteem; mental health challenges; exclusion, etc.

NB to facilitator: it may help, to build an example tree, while you are discussing these points above. You should have example flash cards to put in each part of the tree and ask participants where to put each.

Using the cards you have as a starting point, begin to build your problem trees. Some groups will make a tree for the experience of poor mental health, and the others will make a tree for what hinders mental health services. You will also be given blank cards, if there are things that were not captured in prior focus groups, but that you think are important to consider.

If it helps, you can imagine a person that you know, or that you have heard of, who is living through these issues right now. How would you build a tree to describe their life and experiences? How would you build a tree to describe their quest to seek treatment/support with the things they find difficult?

Instructions: Hand out cards to each group, showing the themes that emerged during the FGDs. Show participants the example problem tree below and give them 1 hour to discuss and create problem trees within their groups. In each group, provide a recorder device to capture the discussions being held by the participants.

Copyright © STARS-C project. Please contact project PI Dr. Rochelle Burgess prior to reproduction/reuse.

For peer review only - http://bmjopen.bmj.com/site/about/guidelines.xhtml

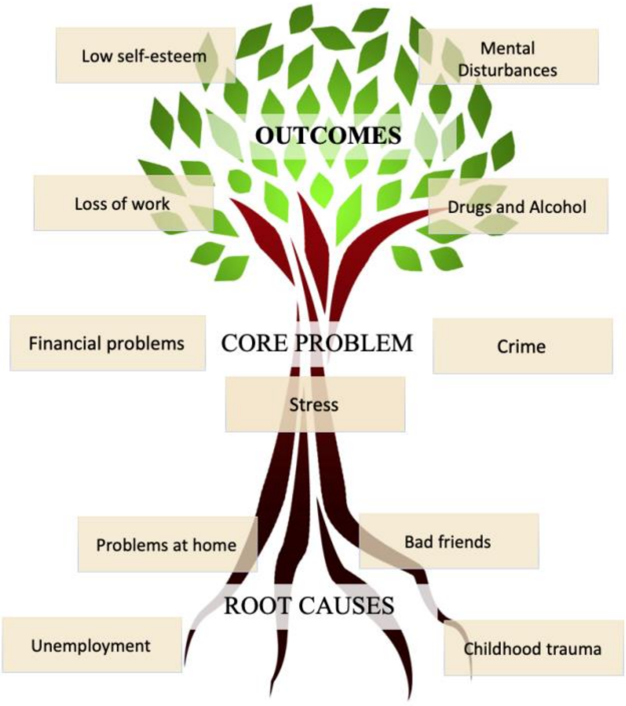

Step 5. After 1 hour, ask a representative from each group to share their problem tree with the rest of the participants (which should take approximately another hour).

Step 6. While the participants share their trees, one facilitator should be taking notes to support later analysis. Another should be taking more general notes to facilitate discussion. Note the similarities and differences between trees, and the challenges and outcomes of healthcare vs health services. These should be shared with the wider group, and participants should be asked for their thoughts on what is being shared.

Session 2
Storytelling of an ideal world – imagining outcomes and outputs

The aim of this session is to identify potential solutions to improve mental health services, and mental health outcomes. This is a long-term plan but should give participants a chance to think about what actions are required to achieve this long-term vision.

Step 1. Facilitators present the following phrase.

"The way we think about the future often focuses on the immediate future. However,when thinking is inspired by a vision, there is more room to achieve things which are thought of as 'unthinkable'. A vision for a better future gives us hope and increases motivation to take action to pursue that vision"

Copyright © STARS-C project. Please contact project PI Dr. Rochelle Burgess prior to reproduction/reuse.

Step 2. Participants should work in the same groups from activity 1.

Script: "Imagine your community 20 years from now. The national television agency (Día a Día/Séptimo Día) has prepared a programme on the outstanding achievements your community has made to increase the rates of access to mental health services and improving mental health in the community. The television/radio programme was prepared based on interviews with community members, local authorities, traditional leaders, and health institutions working in the district. Imagine what the programme would report about your community's achievements in mental health. They have completed a special feature, on two people who have experienced this change. One person is someone whose mental health has been improved, and another is a practitioner who has worked with the patient and the community to build that change.

NB for facilitators: These questions should be handed out to each group on a piece of paper

General questions to consider for all parties

- ❑ What are major changes your community has made in the last 20 years to ensure good mental health in your community?
- ❑ What are the major changes your community has made in the last 20 years to increase the rates of access to mental health services?
- ❑ As a mental health provider, ¿what have you done to improve the mental health of your community?
    - Example: If you are a psychologist, how did you help your community?
- ❑ How have community leaders have supported efforts to address poor mental health?

Questions for your main characters:

- ❑ What actions did you do to start making life changes in terms of your mental health? Who was involved?
- ❑ What action plan did they follow in the first year to make the change happen?
- ❑ How did they convince other people who are important in their lives that this was the right decision?
- ❑ How did they keep going in the long run?

Scrip continued: "in your groups, you will need to write a story about this future world. It may help you to think about the questions in on the attached sheet of paper. You will present your story to the group in a role play (no more than 10 minutes long) of a television interview. There should be four speaking roles:

1) The journalist (who could be asking some of the questions we have provided)
2) a main character who has benefitted from the new world and services (could be the same person you thought about to help you do activity 1)
3) a health care provider
4) A key person who you feel is important to the story. (i.e could be a family member, a community leader, a politician, a friend, etc)

You will have 1 hour to work on this.

Copyright © STARS-C project. Please contact project PI Dr. Rochelle Burgess prior to reproduction/reuse.
For peer review only - http://bmjopen.bmj.com/site/about/guidelines.xhtml

Step **3**. After 1 hour, ask the groups to present their plays within each site. Then ask them to vote for the better story as this will be presented to the broader group including participants from the other site. After deciding which play to present, ask participants to add or improve their stories if they think they should.

Step 4. Finally, let participants present the play from each site to one another. The facilitators should be taking notes and asking people to think about similarities or make comments towards what is being presented after each play. Audio and video record each presentation and the plenary discussion for future analysis.

Session 3
Mapping and intervention building

The aim of this final session is to identify interventions which could be used to improve better mental health and mental health services in communities. It may be worth stating at this stage, that these discussions will shape how we run the second stage of our project – which are the activities we facilitate to improve mental health and improve relationships between mental health services and communities over the course of the next year.

Step 1. Divide participants into same groups as for previous activity

Step 2. Explain that they will need to think back to the problem trees from Activity 1 and what was discussed in Activity 2.

Step 3. Tell participants that they have 1 hour to consider these challenges and imagine possible solutions. Make cardboards with the following questions.

1. What are the interventions you need to improve mental health and mental health services in your community?
2. What resources do you need to implement those interventions/actions?
3. What are the expected outcomes of implementing those interventions?

Step 4: Ask participants to write down the answer to those questions in flashcards and then to paste them under each cardboard.

NB to facilitator: Register the answer provided. If there is enough time, share results with the broader group trying to highlight similarities and differences.

Closing statements

Participants were thanked for their time and contributions. Facilitators went around the room asking about people’s experiences and any feedback for future activities.
Facilitators shared next-steps for the project to have a sense of continuity and stay in touch with the community.

Copyright © STARS-C project. Please contact project PI Dr. Rochelle Burgess prior to reproduction/reuse.
For peer review only - http://bmjopen.bmj.com/site/about/guidelines.xhtml
For peer review only

ToC STARS-C Project

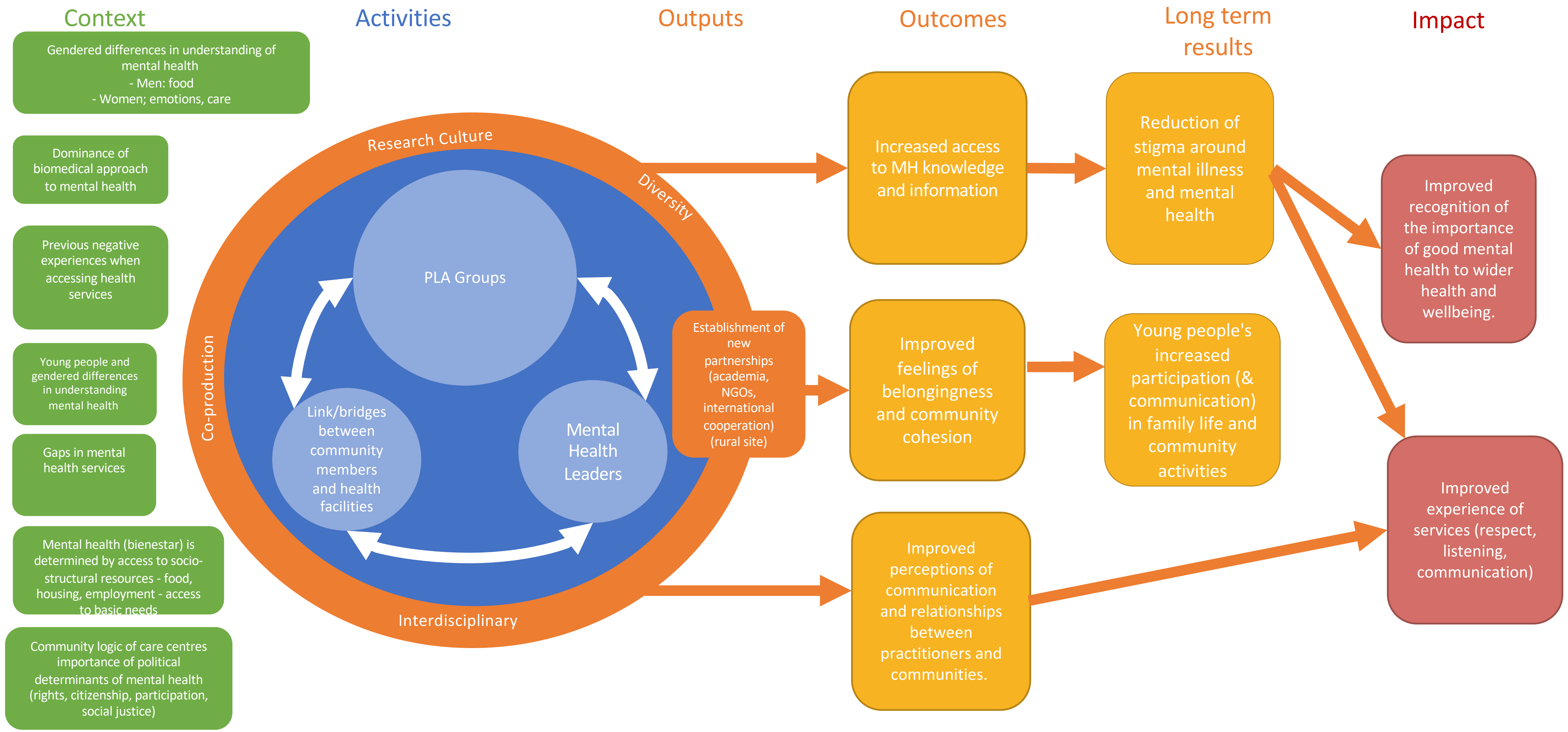

Potential indicators

P 01: Greater acceptance of others seeking treatment
P 01: More positive perceptions of mental illness
P 01: Helping others to seek treatment
P 01 y P 03: Increased willingness to seek treatment
P 02: Increased feelings of community support
P 02: Positive sense of self/identity
P 02: Increased feelings of inclusion and acknowledgement in the community
P 02: Increased sense of attachment to place/home
P 02: percentage of people from the communities who attend community activities organised by the project
P 02: Improved perception of individual and collective agency
P 03: People feel recognised, listened to and acknowledged by health providers and systems
P 03: Increased feelings of citizenship and that rights to quality care are being met

Assumptions

1. Health care providers are willing to attend community led forums, and provide guidance and contribute to community actions to improve mental health, including those informed by social determinants of health, human rights and traditional medicines
2. Local governments are willing to let community members have more ownership over the use of local spaces and environments (urban site)
3. Rural dwellers have reliable access to mental health practitioners and support
4. Young people have the resources and freedom to participate in mental health enhancing activities

For peer review only - http://bmjopen.bmj.com/site/about/guidelines.xhtml
