# Supplementary material for: Using participatory action research to reimagine community mental health services in Colombia: a mixed-method study protocol
Source: BMJ Open. 2022 Dec 21;12(12):e069329. doi: 10.1136/bmjopen-2022-069329 (PMC9772630; doi:10.1136/bmjopen-2022-069329)
Supplement: Reviewer comments [file bmjopen-2022-069329.reviewer_comments.pdf]

PEER REVIEW HISTORY

BMJ Open publishes all reviews undertaken for accepted manuscripts. Reviewers are asked to complete a checklist review form (**http://bmjopen.bmj.com/site/about/resources/checklist.pdf**) and are provided with free text boxes to elaborate on their assessment. These free text comments are reproduced below.

ARTICLE DETAILS

**TITLE (PROVISIONAL)**	Using Participatory Action Research to re-imagine community mental health services in Colombia: A Mixed-Methods Study Protocol
**AUTHORS**	Burgess, Rochelle; Dedios Sanguineti, María Cecilia; Maldonado-Carrizosa, Darío; Fonseca, Laura; Vera San Juan, Norha; Lucumí, Diego; González-Gort, Mónica; Melgar, Mónica Carreño; Gaviria, María Fanny; Tovar, Diego; Jovchelovitch, Sandra

This article was not externally reviewed at BMJ Open. Protocols that have been independently assessed prior to submission to BMJ Open are usually fast-tracked to publication on the grounds that further substantial changes will not be possible. This independent assessment will usually be external, independent review for both a substantial grant award from a non-commercial or government funder and ethics approval.
